# VeGA: A Versatile Generative Architecture for Bioactive Molecules across Multiple Therapeutic Targets

**DOI:** 10.1021/acs.jcim.5c01606

**Published:** 2025-10-02

**Authors:** Pietro Delre, Antonio Lavecchia

**Affiliations:** Department of Pharmacy, “Drug Discovery Laboratory”, 9307 University of Naples Federico II, via Domenico Montesano 49, Naples I-80131, Italy

## Abstract

In this paper, we present VeGA, a lightweight, decoder-only Transformer model for *de novo* molecular design. VeGA balances a streamlined architecture with robust generative performance, making it highly efficient and well-suited for resource-limited environments. Pretrained on ChEMBL, the model demonstrates strong performance against cutting-edge approaches, achieving high validity (96.6%) and novelty (93.6%), ranking among the top performers in the MOSES benchmark. The model’s main strength lies in target-specific fine-tuning under challenging, data-scarce conditions. In a rigorous, leakage-safe evaluation across five pharmacological targets against state-of-the-art models (S4, R4), VeGA proved to be a powerful “explorer” that consistently generated the most novel molecules while maintaining a strong balance between discovery performance and chemical realism. This capability is particularly evident in the extremely low-data scenario of mTORC1, where VeGA achieved top-tier results. As a case study, VeGA was applied to the Farnesoid X receptor (FXR), generating novel compounds with validated binding potential through molecular docking. The model is available as an open-access platform to support medicinal chemists in designing novel, target-specific chemotypes (https://github.com/piedelre93/VeGA-for-de-novo-design). Future developments will focus on incorporating conditioning strategies for multiobjective optimization and integrating experimental in vitro validation workflows.

## Graphical abstract

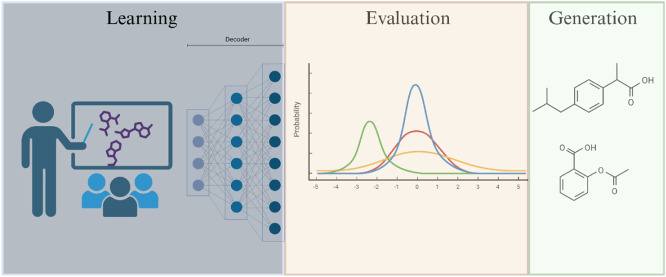

## 1 Introduction

### 1.1 Background

Drug discovery involves navigating a vast and complex chemical space, which is estimated to contain up to 10^23^ synthetically accessible small molecules with potential pharmacological activity.^1^ This space is further constrained when designing compounds that must selectively interact with specific biological targets while meeting multiple drug-like criteria, such as solubility, synthetic accessibility, and favorable pharmacokinetics.^2^ These constraints contribute to the high costs and high failure rates of traditional drug development.^3^ *De novo* molecular design offers a promising alternative, as new chemical structures are generated *de novo*, rather than relying on virtual screening or the optimization of compounds discovered through human intuition.^4,5^ Recent advances in machine learning and deep learning have revolutionized this process, enabling more efficient and data-driven exploration of chemical space.^6–8^ Generating molecules from linear representations, such as the Simplified Molecular Input Line Entry System (SMILES),^9^ has become central in computational drug discovery.^10^ Chemical language models (CLMs) learn the syntax of molecular representations, capturing implicit chemical rules, enabling the generation of novel compounds with desirable pharmacological properties.^11,12^ In this context, Transformer-based architectures, originally developed for natural language processing, have shown excellent performance in molecular generation tasks. While graph-based models ensure strong control over structural validity, recent literature suggests that CLMs are often more efficient at generating large and structurally diverse molecules by better capturing complex features like aromaticity.^7,12^

### 1.2 State of the Art

*De novo* design models span multiple architectures, including recurrent neural networks (RNNs),^13–18^ VAEs,^10,19–21^ GANs,^22–25^ and more recently Transformers.^18,26–28^ As the field has matured, various strategies have been proposed to improve the validity, diversity, and chemical realism of generated SMILES. These include curriculum learning, where models are exposed to progressively more complex structures to improve convergence,^29^ and two techniques to reduce syntactic errors and enhance validity: SMILES augmentation,^30^ which introduces noncanonical representations during training, and chemical symbol tokenization,^31^ an alternative to one-hot encoding. The introduction of attention mechanisms has further advanced molecular generation.^32^ Since 2021, increasingly sophisticated Transformer architectures based on SMILES have enhanced molecular generation and optimization.^33^ For example, LLaMol uses attention to learn long-range dependencies and enables property prediction during fine-tuning,^26^ while MolGPT incorporates conditional generation to design molecules with desired properties.^28^ Among the most relevant recent frameworks is REINVENT 4 (R4), which combines high-performance RNNs with Transformer mechanisms for SMILES-based generation.^18^ More recently, Özçelik et al. introduced the Structured State Space Sequence (S4) model, a state-of-the-art architecture designed to capture efficiently long-range dependencies in sequences.^34,35^ S4 demonstrated strong performance in bioactivity learning and chemical space exploration for designing kinase inhibitors.^36^ In 2025, Nakamura et al. developedTRACER, which combines a conditional Transformer with Monte Carlo tree search to generate optimized molecules with desired properties and synthetically feasible routes.^37^

We selected S4 and R4 as our primary benchmarks because they represent the leading edge of sequence-based generative chemistry. Their complementary architectures, with R4 leveraging recurrent networks and S4 employing a state-space approach, provide a rigorous context for evaluating the specific innovations introduced by VeGA.

### 1.3 VeGA Model

In this paper, we present VeGA, a lightweight decoder-only Transformer model for *de novo* molecular design that integrates state-of-the-art techniques into a computationally efficient framework (Figure 1). VeGA features a significantly leaner architecture compared to many contemporary models,^18,26,37^ reducing the number of parameters while maintaining robust generative performance.

**Figure 1:**
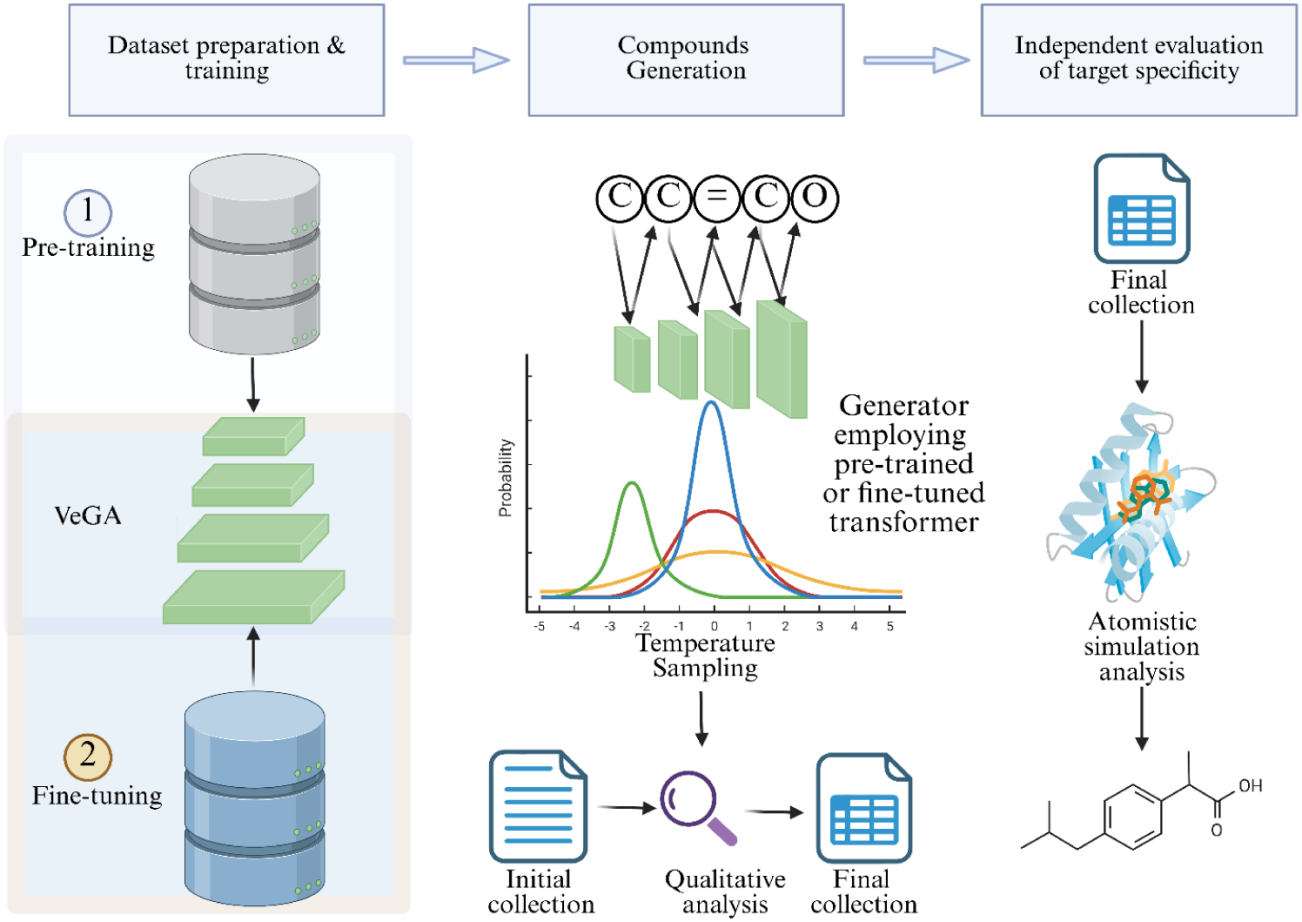
Workflow of the VeGA framework for *de novo* compound generation. The model is first pretrained on large-scale molecular data sets and then fine-tuned on target-specific data. Following compound generation with the pretrained or fine-tuned Transformer, the molecules undergo qualitative analysis and filtering from raw to final collections. Independent evaluation of target specificity includes docking and atomistic simulation. Figure created with BioRender.com.

We demonstrate VeGA’s capabilities across a series of rigorous evaluations. First, in the standardized MOSES benchmark, VeGA ranks among top-performing models, demonstrating its strength as a general-purpose generator. Second, in a direct comparison on a large-scale ChEMBL^38^ generation task, it is competitive with powerful baselines such as S4 and R4, particularly excelling in generating a higher diversity of molecular scaffolds. The model’s versatility is further tested via transfer learning across five pharmacological targets: pyruvate kinase muscle isoform 2 (PKM2),^39^ mitogen-activated protein kinase 1 (MAPK1),^40^ glucocerebrosidase (GBA),^41^ mechanistic target of rapamycin (mTORC1),^42^ and farnesoid X receptor (FXR)^43,44^ with data sets containing between 77 and 882 compounds. In this challenging, leakage-safe benchmark, VeGA consistently generates the most novel molecules and shows superior data efficiency in scenarios with limited data. Finally, to illustrate real-world applicability, we conducted a prospective in silico study on FXR, where VeGA generated novel candidate ligands validated via molecular docking. To support reproducibility and encourage community use, we provide VeGA′s full implementation, pretrained models, and comprehensive documentation in an open-source GitHub repository (https://github.com/piedelre93/VeGA-for-de-novo-design).

## 2 Methods

### 2.1 Model Development

We implemented a modular, end-to-end pipeline for *de novo* molecular generation based on an autoregressive Transformer architecture. The pipeline was implemented in Python 3.9 with RDKit 2022.03 ^45^ and TensorFlow 2.9.0.^46^

### 2.2 Data Set Preparation

The training set (TS) was curated from the ChEMBL28 database.^38^ The workflow, implemented in KNIME^47^ using RDKit,^45^ CDK,^48^ and Open Babel nodes,^49^ involved the following steps:

(i) discarding compounds without SMILES notation;
(ii) removing stereochemistry;
(iii) desalting and neutralizing compounds;
(iv) excluding inorganic compounds and those containing metal atoms;
(v) excluding compounds containing elements other than H, C, N, O, F, Br, I, Cl, P, or S;
(vi) converting all entries to neutralized canonical SMILES using Open Babel;
(vii) removing duplicates;^50^
(viii) discarding SMILES strings in the bottom or top 5% of the character length distribution.

The final TS consists of 1,092,285 compounds (ChEMBL-DB). Each SMILES string in ChEMBL-DB was sanitized with RDKit,^45^ and kekulization was applied to eliminate aromaticity ambiguities. A regex-based tokenizer was used to split each cleaned SMILES into chemically meaningful tokens. Sequences with fewer than three tokens or exceeding the 99th percentile of token length were excluded (computed over the full data set) to avoid excessively long inputs. Additionally, a data set of high affinity FXR ligands (FXR-DB) was curated by extracting affinity entries from ChEMBL28^38^ labeled with the FXR Target ID (ChEMBL2047). The FXR-DB was filtered by retaining entries: annotated exclusively with EC_50_ < 1 μM, from human targets (“target_organism” = “*Homo sapiens*”), annotated as direct binding (“assay_type” = “B”), and without warnings in the “data_validity_comment” field. The final FXR-DB contained 882 compounds after desalting, neutralizing, and removing duplicates. Other curated data sets: COCONUT, GBA, MAPK1, mTORC1, and PKM2, containing 32,000, 132, 246, 77, and 436 SMILES respectively, were retrieved from https://zenodo.org/records/12666371.^36^ SMILES strings were tokenized using a chemically informed, atom-wise approach adapted from Schwaller et al.,^31,51^ decomposing sequences into meaningful chemical substructures (e.g., atoms, bonds, branches). Token coverage was validated by successfully parsing and reconstructing >99% of SMILES in the data sets from their tokenized sequences.

### 2.3 Hyperparameter Tuning

To establish the optimal architecture for the generative Transformer model, we conducted a systematic hyperparameter tuning using the Optuna framework with a Bayesian optimization strategy (Tree-structured Parzen Estimator, TPE).^52,53^ To balance computational efficiency and representativeness, the optimization was performed on a subset of 50,000 SMILES randomly sampled from ChEMBL-DB. This subset was split into training (90%) and validation (10%) sets for the optimization trials. The search space included embedding dimensionality ([60–120], step size: 20), number of Transformer layers ([1–4], step size: 1), number of multihead attention units ([1–4], step size: 1), dropout rate (0.1–0.3, step size: 0.05), Feed-Forward Network (FFN) dimensionality ([100–500], step size: 100), and batch size ([32, 64, 128]). Optimization was conducted over 100 trials, with each trial using a reduced training schedule (up to 20 epochs) and early stopping. The configuration yielding the lowest validation loss was selected for final training. Validation loss was computed as masked sparse categorical cross-entropy over nonpadding tokens, reflecting the model’s ability to reconstruct valid chemical sequences. Optimized hyperparameters and performance across trials are shown in Figure S1.

### 2.4 Model Architecture

VeGA is a decoder-only Transformer with unidirectional left-to-right autoregressive decoding, generating one token at a time conditioned on previously generated tokens. It consists of four layers of masked multihead self-attention (four heads per layer), a feed-forward network of 300 units, an embedding dimension of 100, and 15% dropout (Figure S2 and Table S1). The attention matrix is defined as in Vaswani et al.:^54^

$$Attention\left(Q,K,V\right)=soft\max\left(\frac{QK^{T}}{\sqrt{d_{k}}}+M\right)V$$

Each term is as follows: Attention­(Q, K, V) produces a contextual representation by combining Query (Q), Key (K), and Value (V) matrices. The equation also includes a scaling factor $\left(\sqrt{d_{k}}\right)$ to stabilize gradients during training and a Masking matrix (M).

Positional information was incorporated using dynamic positional encodings, a technique popularized by the original Transformer architecture. Unlike learnable positional embeddings, this method uses a fixed, deterministic function based on sine and cosine waves to assign a unique vector to each position in the sequence. This approach provides two key advantages:

- Generalization to unseen sequence lengths: The encoding is functional rather than learned, allowing positional information for sequences of any length. This is crucial for molecular generation, where novel compounds may exceed lengths seen during training.
- Parameter efficiency: This method adds no trainable parameters, contributing to VeGA’s lightweight design and computational efficiency.^55–57^
$$PE_{\left(pos,2i\right)}=\sin\left(\frac{pos}{10000^{2i/d_{\mathrm{mod}el}}}\right),PE_{\left(pos,2i+1\right)}=\cos\left(\frac{pos}{10000^{2i/d_{\mathrm{mod}el}}}\right)$$

Each term is described as *PE*_(*pos*,2*i*(+1))_ assigns unique vectors to token positions; *pos* is the token position; *i* is the embedding dimension index; *d_model_* is the embedding dimensionality (100 in VeGA); sin/cos create unique position patterns preserving relative distances.

The model was trained using the Adam optimizer with a customized learning rate scheduler.^55–57^

$$lr\left(t\right)=d_{model}^{-0.5}\times \min\left(t^{-0.5},\;t\times warmup\_steps^{-1.5}\right)$$

Each term is described as *lr*(*t*) is the learning rate at step t; *d_model_* is the embedding dimensionality (100); *t* is the current training step; *warmup* _ *steps* is the number of steps in initial warmup; min­() selects the smaller value between two arguments to control warmup-to-decay transition.

ChEMBL-DB was randomly split into 80% training and 20% validation subsets, stratified by molecular length to preserve structural diversity. The model was optimized using a masked sparse categorical cross-entropy loss function. A binary mask excluded padding tokens (“<PAD>”) from loss computation, ensuring that only active tokens contributed to gradients. As a result, the reported loss corresponds to the average cross-entropy calculated exclusively over active (nonpadding) tokens in each batch. Training proceeded for up to 300 epochs, with continuous monitoring of training and validation loss to prevent overfitting and ensure stable convergence. A learning rate reduction callback was activated if validation loss plateaued for a predefined number of epochs. To ensure robust training, we implemented a 2-fold strategy to manage batch composition and preserve diversity. First, we employed a length-based batch balancing approach: sequences of similar length were grouped to minimize padding and improve computational efficiency; batch order was randomized each epoch to avoid length-associated biases. Second, to enhance syntactic diversity, we used SMILES augmentation,^30^ where canonical SMILES were replaced with randomized, noncanonical, syntactically valid and chemically equivalent representations with a 10% probability during training. This probability was selected as it balances structural diversity (Internal Diversity) and chemical realism (Fréchet ChemNet Distance) (Table S2). These strategies were used alongside early stopping and learning rate scheduling. Experiments were conducted on a workstation with an NVIDIA RTX A2000 GPU (12 GB VRAM) and 12 GB system RAM.

### 2.5 Molecular Generation

Novel molecules are generated via a stepwise probabilistic process. The model constructs SMILES strings autoregressively, predicting each new token based on the preceding sequence. The probability of a sequence (S) is defined as

$$P\left(S\right)=\overset{n}{\underset{i=1}{\prod }}P\left(s_{i}|s_{1},s_{2},...,s_{i-1}\right)$$

Generation used causal attention to preserve valid conditional dependencies, as described in Vaswani et al.:

$$A\left(Q,K,V\right)=\mathrm{softmax}\left(\frac{QK^{T}}{\sqrt{d_{k}}}+M\right)V$$

The process is further modulated by a temperature-controlled sampling strategy, which balances exploitation of learned chemical patterns with exploration of novel structures:

$$P_{\tau }\left(s_{i}|s_{1},...,s_{i-1}\right)=\mathrm{softmax}\left(\frac{z_{i}}{\tau }\right)$$

Generation terminated when an end-of-sequence token ($) was sampled or a maximum length was reached. All generated strings are validated for chemical correctness and standardized to canonical SMILES using RDKit.

### 2.6 Transfer Learning for Target-Specific Generation

To adapt the model for target-specific molecular design and rigorously evaluate generalization, a leakage-safe holdout set was constructed by splitting active compounds by Bemis-Murcko scaffolds and applying sphere exclusion: any test-set molecule with Tanimoto similarity ≥ 0.6 (ECFP4, 2048 bits) to a training-set molecule was removed. The remaining molecules constituted the final holdout set. Subsequently, the base VeGA model, pretrained on ChEMBL, was fine-tuned using only the training portion of the split data, while the holdout portion was kept separate for subsequent evaluation. The pretrained VeGA model was fine-tuned exclusively on the training portion, with the holdout set reserved for evaluation. Fine-tuning used up to 100 epochs, a reduced learning rate of 5e-5, a batch size of 32, and early stopping based on validation loss to prevent overfitting on smaller target-specific data sets.

### 2.7 Evaluation

#### 2.7.1 Quality Metrics

To assess the quality of the generated molecules, we employed a comprehensive suite of metrics to evaluate performance, from basic chemical correctness to advanced measures of novelty and distributional fidelity. All metrics were computed using RDKit and in-house Python scripts.

Quality Metrics:^45^

- Validity: Percentage of SMILES strings generated that represent chemically valid molecules.
- Uniqueness: Percentage of unique molecules within the set of validly generated compounds.
- Novelty: Percentage of unique, valid molecules that are not present in the training set.

Physicochemical and Distributional Metrics:^58^

- Physicochemical properties: distributions of molecular weight (MW), logP, number of rings, hydrogen bond donors (HBD), hydrogen bond acceptors (HBA), and rotatable bonds were analyzed. Drug-likeness was also assessed using Lipinski’s Rule of 5 and the Pan-Assay Interference Compounds (PAINS) filter.
- Drug-likeness Scores: We calculated two standard drug-likeness metrics: the Quantitative Estimate of Drug-likeness (QED),^59^ ranging from 0 to 1, and the Synthetic Accessibility (SA) score,^60^ where lower values indicate easier synthesis.
- Distributional Similarity: To quantify similarity between property distributions of generated and reference molecules, we applied:
  Kolmogorov–Smirnov (KS) test: measures the maximum pointwise difference between cumulative distribution functions.
  Kullback–Leibler (KL) Divergence: measures the average divergence between two probability distributions.

- Fréchet ChemNet Distance (FCD): A standard metric assessing distributional similarity between generated and real molecules in a learned chemical space.^61^ To mitigate the “size trap” phenomenon, which arises from differences in set size, we randomly sampled equally sized subsets from both reference and generated databases. This process was repeated three times for statistical robustness.^62^

Target-specific and novelty-focused metrics:

- Recovery Rate (RR): proportion of holdout molecules rediscovered in the generated chemical space. A holdout molecule is considered recovered if its maximum Tanimoto similarity to any generated molecule is ≥ 0.6 on extended connectivity fingerprints. This threshold follows a recently published protocol.^63^
- Scaffold Recovery (RR_scaffold): Fraction of unique Bemis-Murcko scaffolds from a data set present in the generated set.
- Similarity to Nearest Neighbor (SNN): Average Tanimoto similarity between each generated molecule and its nearest neighbor in the training set.^63^
- Internal Diversity (IntDiv): Average pairwise Tanimoto dissimilarity within the generated set.

#### 2.7.2 Scaffold Diversity Analysis

To quantitatively compare structural diversity among VeGA, S4, and R4, we performed a scaffold-based analysis on sets of molecules generated by each model:^36^

1. Scaffold Extraction: Bemis-Murcko scaffolds were extracted with RDKit, and only unique scaffolds were retained for nonredundant analysis.
2. Scaffold Clustering: Unique scaffolds were converted into Morgan fingerprints (radius 3, 2048 bits) and clustered using a greedy algorithm with a Tanimoto similarity threshold of 0.6. Each scaffold was assigned to the first cluster with similarity ≥ 0.6; otherwise, it initiated a new cluster.
3. Diversity Metrics:
  a. Distinct Scaffolds: Number of clusters generated, reflecting distinct scaffold families.
  b. Scaffold Diversity Index (SDI): Shannon entropy of the cluster population distribution, where higher values indicate broader scaffold variety and more uniform representation.

#### 2.7.3 Standardized Benchmarking with MOSES

To contextualize VeGA’s performance and ensure fair comparison with established methods, we benchmarked the model using the Molecular Sets (MOSES) framework.^64^ Following the standard protocol, we trained a new instance of VeGA from scratch on the MOSES training data set and generated 100,000 molecules for evaluation. Performance was assessed with MOSES metrics, including validity, uniqueness, novelty, FCD, and two measures of internal diversity. Specifically, we calculated IntDiv1, the average pairwise Tanimoto dissimilarity, and IntDiv2, a stricter variant applying a squared average to penalize clusters of highly similar molecules. This analysis, presented in the Results and Discussion section, enables a direct and unbiased comparison of VeGA with diverse state-of-the-art generative architectures.

#### 2.7.4 Chemical Space Visualization

To qualitatively assess chemical space coverage, dimensionality reduction and visualization were performed using the Uniform Manifold Approximation and Projection (UMAP) algorithm. For each target, the training set, holdout set, and 10,000 generated molecules were converted into Morgan fingerprints (ECFP4, 2048 bits, radius 2). UMAP was fitted on the combined fingerprints of the training and generated sets to learn a 2D projection, after which holdout set coordinates were transformed into this space. This ensured accurate representation of holdout molecules within the chemical space explored by the model. UMAP was implemented via the umap-learn library using the Jaccard metric, 30 neighbors, and a minimum distance of 0.1.

### 2.8 Molecular Docking Methodology

Generated focused libraries were docked into the FXR crystal structure (PDB code: 3DCT).^65^ The protein structure (.pdb file) was preprocessed using the Protein Preparation Workflow in the Schrödinger Suite 2024–1 ^66^ to add missing hydrogen atoms, rebuild incomplete residues and rings, and assign appropriate protonation states at physiological pH. Ligands were prepared using LigPrep^67^ enumerating relevant tautomers and protonation states at pH 7.0 ± 2.0. All possible enantiomers of each ligand were also generated. Docking simulations were performed using the Grid-based Ligand Docking with Energetics (GLIDE) protocol,^68^ implemented in Schrödinger Suite 2024–1.^69^ Full ligand flexibility was allowed, while the receptor was held rigid. The OPLS_2005 force field^70^ was applied with standard precision (SP) docking mode. A cubic grid was centered on the cognate ligand, with inner box dimensions of 10.00 Å and outer box dimensions of 30.00 Å. Docking reliability was confirmed by redocking the original ligand into the binding site, yielding a root-mean-square deviation (RMSD) of 0.81 Å (heavy atoms only), which supports the robustness of the docking protocol.

## 3 Results and Discussion

As a result of hyperparameter tuning (Figure S1), VeGA features an embedding dimension of 100, 4 attention heads, 4 layers, a dropout rate of 0.15, and a feed-forward network of 300 units (Figure S2). Unlike large-scale models such as TRACER,^37^ LLaMol,^26^ or MolGPT,^28^ which require substantial computational resources and are often optimized for conditional or prediction tasks, VeGA fills the gap for compact, interpretable autoregressive models tailored for SMILES-based molecular generation and efficient fine-tuning on small data sets. As detailed in Table S5, VeGA comprises only ∼0.8 million trainable parameters, which is between one and 2 orders of magnitude lower than MolGPT (∼8.6 M), LLaMol (∼15 M), and TRACER (∼45 M), while achieving an average inference time of ∼20 ms per molecule on a single workstation GPU. These quantitative benchmarks, together with markedly lower hardware and training-time requirements, highlight VeGA′s computational efficiency and accessibility.

To rigorously evaluate VeGA, we implemented a multistage validation strategy that systematically supports the case for VeGA as a versatile and efficient generative model.

First, we benchmarked VeGA on the standardized MOSES framework to establish its performance as a general-purpose generator (Section 3.1). Second, we conducted a detailed comparative analysis of its unconditional generation capabilities on the large-scale ChEMBL database^38^ by comparing the performance against two well-known models in the literature taken as references: S4 and R4.^18,36^ (Section 3.2).

Next, to test architectural versatility, we evaluated VeGA’s ability to capture the complex structural and chemical syntax of natural products (NPs), a domain structurally distinct from drug-like molecules (Section 3.3). The core of our investigation focused on VeGA’s performance in target-specific applications. We performed a rigorous, leakage-safe evaluation across five diverse pharmacological targets, comparing its ability to capture bioactivity against the S4 and R4 models (Section 3.4).

Finally, to demonstrate practical applicability, we conducted a prospective in silico case study on the Farnesoid X receptor (FXR), validating VeGA-generated ligands for binding potential via molecular docking simulations (Section 3.5).^43,44^

### 3.1 Standardized Benchmarking on MOSES

To establish VeGA’s performance as a general-purpose molecular generator, we first evaluated its unconditional generation capabilities using the MOSES framework.^64^

The benchmark results (Table 1) show that VeGA achieves competitive performance, generating chemically valid (96.6%) and fully unique (100.0%) molecules. A direct comparison with S4 highlights a balance between distributional fidelity and novelty: S4 achieves near-perfect fidelity to the reference distribution with an FCD score of 0.0, whereas VeGA achieves significantly higher novelty (93.6% vs S4’s 88.1%). This higher novelty indicates VeGA’s stronger capacity to explore uncharted chemical space while preserving distributional fidelity (FCD/Test = 0.184). Compared to other architectures, VeGA’s novelty also surpasses that of MolGPT (79.7%), and its FCD score of 0.18 is superior to that of the graph-based JTN-VAE. Furthermore, VeGA’s Internal Diversity (0.86) matches the reference test set, confirming that VeGA maintains realistic chemical diversity alongside novelty (see Table 1 for metric definitions and results). Overall, these results establish VeGA as a generator that balances novelty exploration with strong chemical realism.

**Table 1:** Performance of vega on the MOSES Benchmark^a^

Model	Valid (%)	Unique@1k (%)	Unique@10k (%)	Novelty (%)	IntDiv	IntDiv2	FCD Test	FCD TestSF
Train	n.a.	n.a.	n.a.	n.a.	0.85	0.85	0.01	0.47
HMM	57.60	62.30	56.70	99.90	0.85	0.84	24.46	25.41
NGram	23.80	97.40	92.20	96.90	0.87	0.86	5.51	6.23
Combinatorial	100.00	99.80	99.10	98.80	0.87	0.87	4.24	4.51
CharRNN	97.50	100.00	99.90	84.20	0.86	0.85	0.07	0.52
AAE	93.70	100.00	99.70	79.30	0.86	0.85	0.56	1.06
VAE	97.70	100.00	99.80	69.50	0.86	0.85	0.10	0.57
JTN-VAE	100.00	100.00	100.00	91.40	0.86	0.85	0.40	0.94
LatentGAN	89.70	100.00	100.00	95.00	0.86	0.85	0.30	0.83
VeGA	**96.57**	**100.00**	**100.00**	**93.60**	**0.86**	**0.85**	**0.18**	**0.68**
MolGPT	99.40	100.00	100.00	79.70	0.86	0.85	0.07	0.51
S4	98.40	100.00	100.00	88.10	0.86	NA	0.01	0.43

a Abbreviations: Valid, Validity; Unique@1*k*/10k, Uniqueness at 1k and 10k samples; IntDiv, Internal Diversity; FCD Test, Fréchet ChemNet Distance against the test set; FCD TestSF, Fréchet ChemNet Distance against the scaffold test set.

### 3.2 Comparative Analysis of Unconditional Generation on chembl

To assess performance on a large-scale data set, we conducted a comprehensive benchmark of VeGA against S4 and R4 using ChEMBL. The results (Figure 2) show that VeGA achieves highly competitive outcomes across multiple metrics: validity of 95.26%, uniqueness greater than 99.8%, and novelty of 99.28%. Its drug-likeness scores, including QED (0.56) and SA (2.77), are favorable and comparable to leading models. Analysis of physicochemical property distributions (Figure 3) demonstrates strong alignment with the ChEMBL reference set (Table S3). This similarity was quantitatively confirmed by both the KS test and KL Divergence, which yielded low statistic values, further confirming high distributional fidelity (Table S4). FCD scores further demonstrate good coverage of the reference chemical space across all three models: S4 achieved the best score (0.88 ± 0.03), while VeGA’s score was also competitive (0.97 ± 0.02). The chemical relevance of VeGA’s outputs is supported by the fact that 5.71% of its generated molecules are present in PubChem, despite being absent from the training set (Figure S3). However, VeGA’s primary advantage lies in its superior scaffold diversity. VeGA generated 69,921 distinct scaffolds, surpassing both S4 (63,384) and R4 (62,250), and achieved the highest scaffold diversity index (SDI = 11.10). This ability to explore structurally novel chemical space is critical for lead identification campaigns and highlights one of VeGA’s key strengths. Another key advantage is computational efficiency. VeGA features a parameter count of under one million, making it substantially more compact than S4 and R4. A direct comparison (Table S5) shows that S4 required an extensive 10-day hyperparameter search on multiple high-end GPUs, whereas VeGA’s Bayesian hyperparameter search completed in ∼ 36 h, and pretraining took an additional ∼ 7 h on a single workstation GPU. These results underscore VeGA as both a competitive and accessible solution for drug discovery workflows, particularly in resource-limited environments.

**Figure 2:**
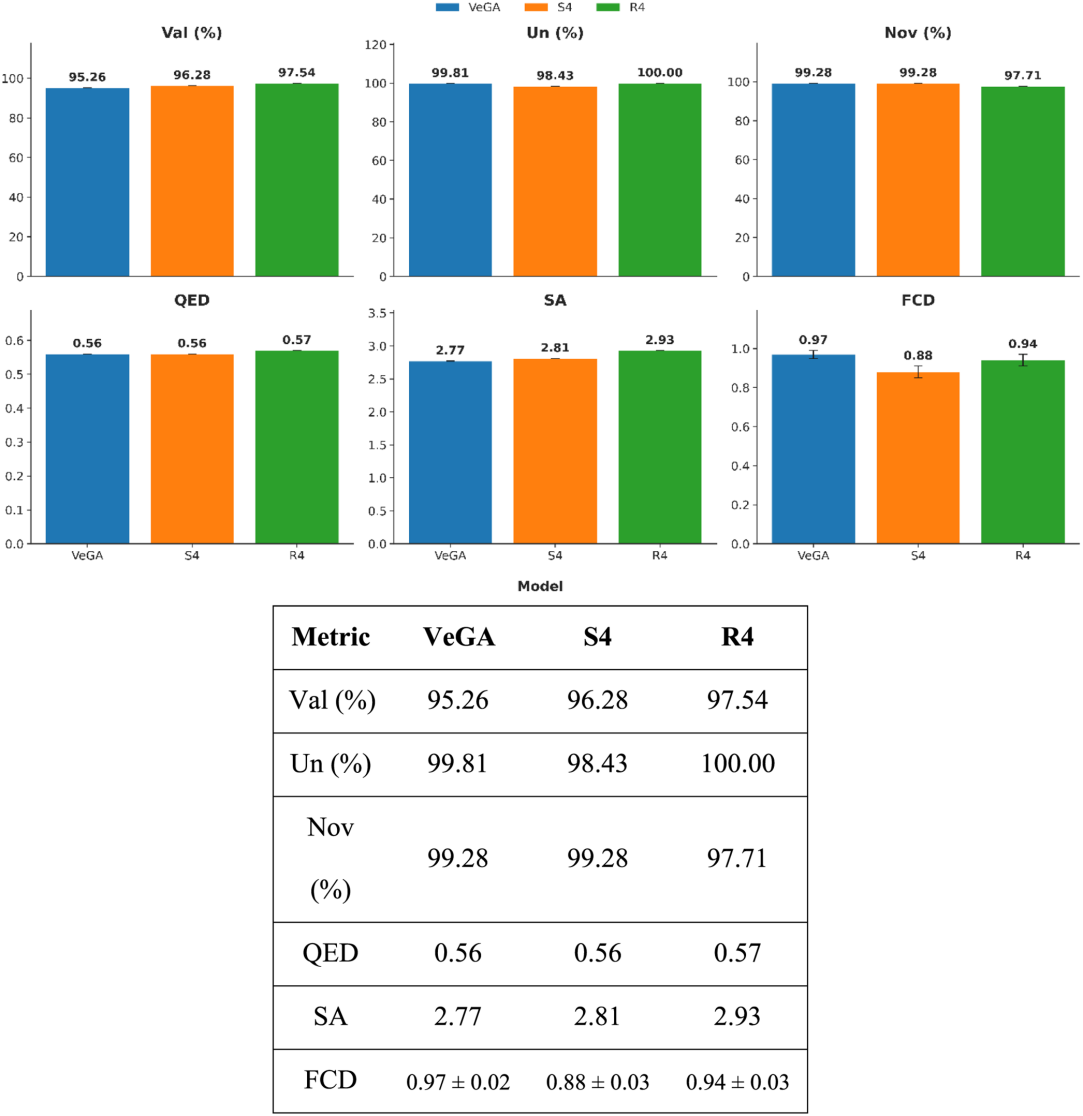
Comparative analysis of key generative metrics for VeGA, S4, and R4. The bar charts report Validity (Val %), Uniqueness (Un %), Novelty (Nov %), Quantitative Estimate of Drug-likeness (QED), Synthetic Accessibility (SA) score, and Fréchet ChemNet Distance (FCD). The results highlight VeGA’s robust ability to generate valid and novel molecules with favorable drug-like properties, comparable to state-of-the-art baselines.

**Figure 3:**
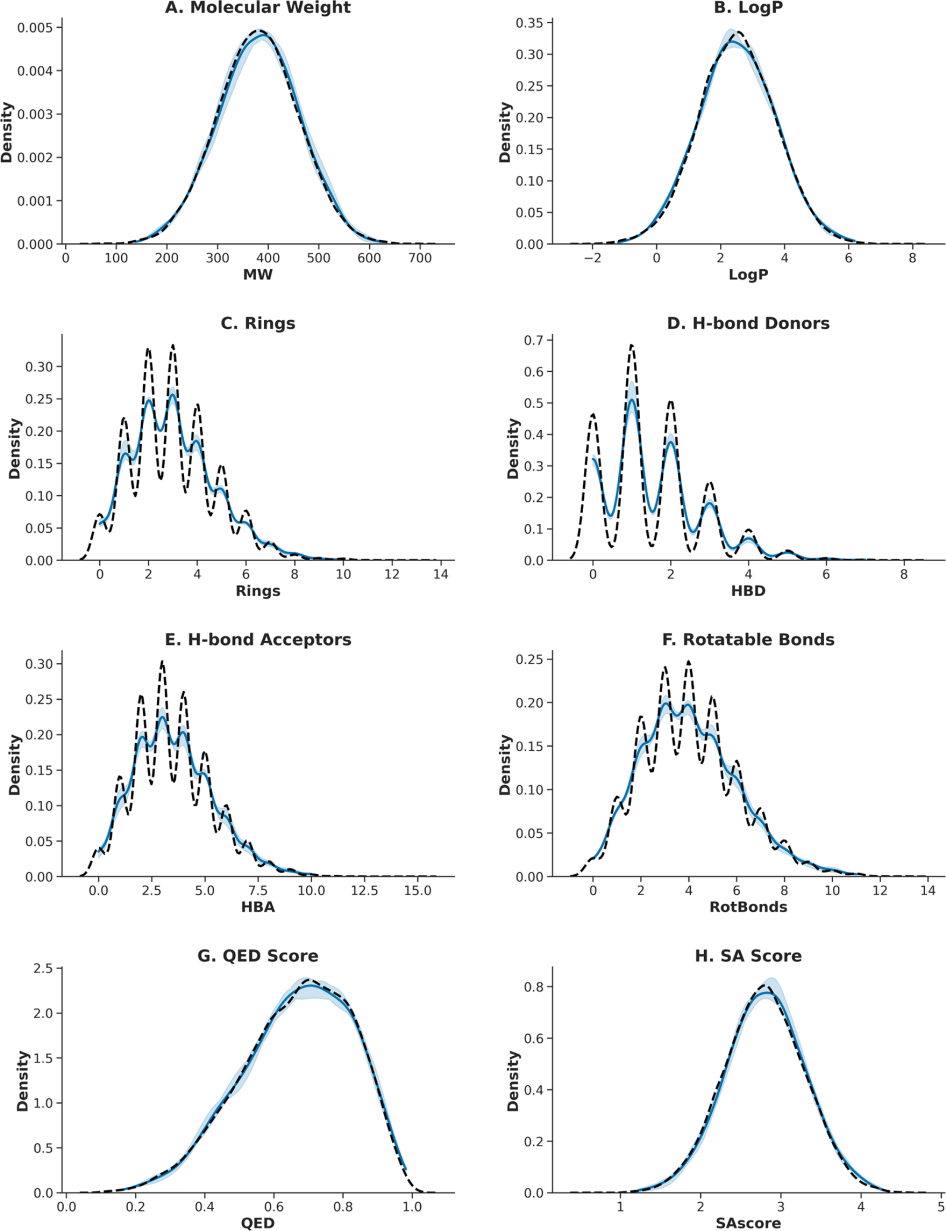
Distributional fidelity of molecules generated by VeGA, S4, and R4. The figure compares key physicochemical property distributions against the ChEMBL reference set. Panels show: (A) Molecular Weight, (B) LogP, (C) Ring Count, (D) H-Bond Donors, (E) H-Bond Acceptors, (F) Rotatable Bonds, (G) QED Score, and (H) SA Score. The close overlap between generated molecules (blue) and reference data (black) confirms the models’ ability to accurately reproduce the characteristics of the target chemical space.

### 3.3 NP Generation

To assess the versatility of our architecture, we tested its ability to learn the syntax of NPs and generate novel, NP-like molecules. This task is particularly challenging, as NPs feature complex topologies and long-range dependencies. For this experiment, we trained a new instance of VeGA on 32,360 structures from the COCONUT database.^71^ Owing to the expanded chemical space, the model automatically created a larger vocabulary of 53 unique tokens (Table S6), demonstrating its flexibility to adapt to domain-specific chemistries. To address the increased complexity, we reduced the sampling temperature to 0.6 during generation.

The base VeGA model achieved 65.0% validity, 68.2% uniqueness, and 96.3% novelty. While this validity is lower than values reported for larger models like S4 (82.6%), VeGA compensates with substantially higher novelty (96.3% vs 40%) and uniqueness (68.2% vs 50%).^36^ This outcome aligns with VeGA’s design philosophy of prioritizing a lightweight, parameter-efficient architecture. To further examine this, we trained a deeper version of VeGA with increased representational capacity. The architectural changes are detailed in the Supporting Information (Table S7).

The comparative evaluation (Table 2) confirms that the deeper model markedly improves validity (+12.3% points), while maintaining high novelty and uniqueness. This demonstrates that structurally complex NPs benefit from models with greater representational power, supporting the hypothesis that greater architectural depth enhances NP generation.

**Table 2:** Performance of Original and Deeper vega Models on NP Generation

Metric	Original VeGA	Deeper VeGA
Validity	65.10%	**77.31%**
Uniqueness	68.22%	**73.51%**
Novelty	96.33%	**97.71%**

A comprehensive evaluation of structural features was also performed (Tables S8 and S9). The NP-likeness score,^72^ computed using RDKit, showed strong agreement between generated molecules and the COCONUT training set (mean 1.58 vs 1.59; KS statistic 0.07), confirming that VeGA successfully captured NP-like chemical patterns (Figure 4).

**Figure 4:**
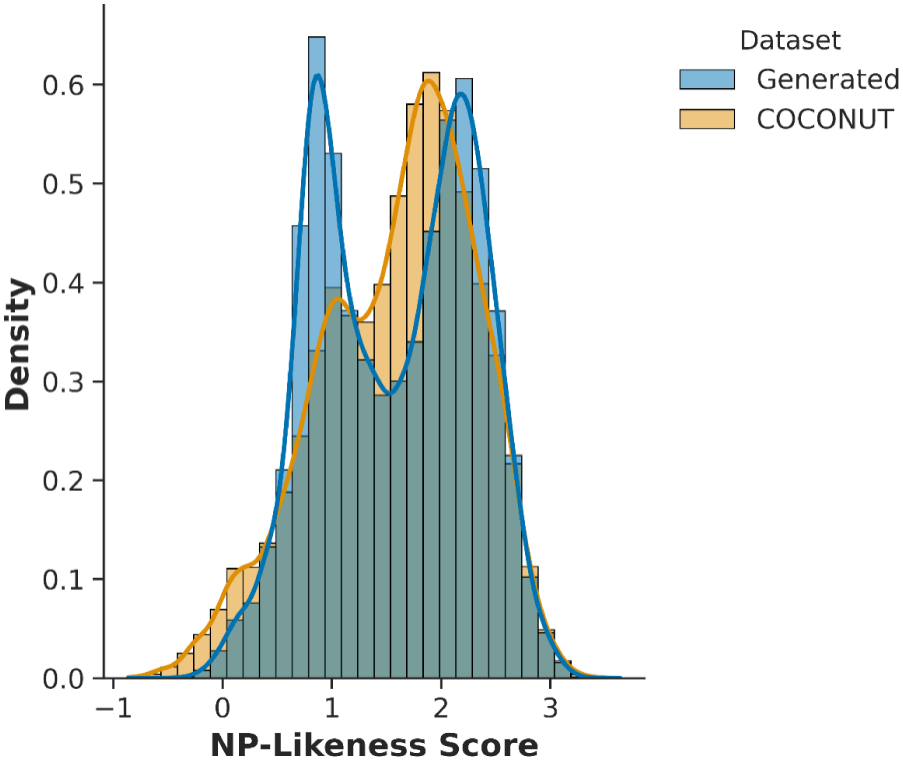
Capture of natural product-like chemical features by VeGA. Distribution of NP-likeness scores for molecules generated by the NP-tuned VeGA model compared with the COCONUT reference database. The near-perfect overlap between the two distributions (means of 1.58 vs 1.59) validates VeGA’s ability to learn and reproduce the defining patterns of this complex molecular class.

This analysis demonstrates that the VeGA framework can handle this challenging domain. Although a full hyperparameter optimization for NP generation remains future work, the results validate that performance scales with architectural capacity.

For rigorous statistical validation, we compared distributions of key structural features using the same properties as Özçelik et al.^36^ KS tests revealed consistently low statistics across most properties (Tables S8 and S9), indicating no significant differences. This broad agreement provides strong quantitative evidence that the model effectively captured defining NP chemical patterns.

### 3.4 Capturing Bioactivity

The ultimate test for a generative model in drug discovery is its ability to explore novel chemical space while successfully rediscovering active molecules for a specific biological target. To assess this, we employed a rigorous leakage-safe holdout protocol (Methods). For each fine-tuned model (VeGA, S4, and R4), we generated 10,000 molecules and calculated a suite of performance metrics. The comparative results (Table 3) contextualize the models’ performance within the chemical space topology, defined as “continuous” when the holdout is close to the training set (maximum Tanimoto similarity just below 0.6) or “disjointed” when more distant (most holdout molecules with Tanimoto similarity <0.4–0.5). This topology directly influences which generative strategy proves most effective. For FXR, characterized by a continuous space, the conservative interpolation strategy of S4 is most effective, achieving the highest molecule discovery rate on the training set (RR_T = 97.40%), scaffold recovery on the holdout set (RS_H = 25.00%), and an excellent FCD score. In contrast, in more disjointed spaces, the models exhibit distinct strengths. R4 demonstrates robustness as an explorer, achieving the highest discovery rate for the challenging PKM2 target (RR_T = 88.10%). S4 also performs strongly in some of these cases, leading on GBA and MAPK1. In this landscape, VeGA’s unique profile emerges. While not always the top performer in raw discovery rates, its strength lies in superior novelty. Across the most disjointed spaces (MAPK1, PKM2, mTORC1), VeGA consistently generates molecules most distant from the training set, as indicated by the lowest SNN scores (definitions of metrics are reported in Table 3 footnotes). This highlights VeGA’s stronger tendency to explore and discover new chemotypes. UMAP projections visually confirm this behavior: VeGA-generated molecules successfully envelop holdout molecules for these targets (Figure S4). Furthermore, VeGA maintains high-quality outputs, achieving the best FCD score on the extremely data-scarce mTORC1 target. In conclusion, this evaluation reveals a nuanced performance landscape: S4 excels as an interpolator in dense spaces, R4 is a robust performer across multiple targets, and VeGA distinguishes itself as the most effective explorer, consistently prioritizing novelty. This positions VeGA as a valuable tool for de novo design campaigns, where the objective is not simply to replicate known scaffolds, but to discover novel chemical matter and new intellectual property.

**Table 3:** Performance of vega, S4, and R4 on Leakage-Safe Holdout Evaluation^a^

Target	Data Split (Train/Holdout)	Chemical Space Profile	Model	Val	Uni	RR_T	RS_T	RS_H	SNN	FCD ± SD
FXR	702/40	Continuous	VeGA	94.30	46.64	96.60	57.04	20.80	0.63	3.95 ± 0.15
			R4	99.10	42.18	96.20	55.63	16.70	0.56	8.34 ± 0.24
			S4	99.40	23.37	97.39	63.37	25.00	0.75	3.60 ± 0.32
GBA	104/24	Disjointed	VeGA	93.23	82.00	68.18	74.36	10.50	0.37	21.43 ± 0.53
			R4	99.04	58.28	76.52	80.77	10.50	0.36	25.70 ± 1.11
			S4	98.00	30.48	78.03	89.74	0.00	0.52	20.24 ± 0.76
MAPK1	197/49	Disjointed	VeGA	94.30	88.83	62.60	48.91	7.00	0.38	13.71 ± 0.32
			R4	99.28	54.33	69.92	59.24	4.70	0.40	15.09 ± 0.53
			S4	98.20	65.94	73.92	66.30	9.30	0.46	13.70 ± 0.40
mTORC1	62/15	Disjointed	VeGA	94.30	89.43	75.32	81.63	16.70	0.38	28.07 ± 1.39
			R4	98.50	72.90	81.82	90.50	16.70	0.32	34.92 ± 1.87
			S4	98.20	46.66	84.42	89.50	16.70	0.48	29.78 ± 1.52
PKM2	348/81	Disjointed	VeGA	92.40	89.64	46.56	52.48	29.90	0.41	9.46 ± 0.22
			R4	99.02	55.12	88.07	90.50	29.90	0.46	8.72 ± 0.33
			S4	98.20	72.19	80.71	78.52	20.90	0.48	9.10 ± 0.31

a Val, validity; Uni, uniqueness; RR_T, recovery rate from the training set; RS_T, scaffold recovery from the training set; RS_H, scaffold recovery from the holdout set; SNN, similarity to nearest neighbor in the training set; FCD, Fréchet ChemNet Distance.

### 3.5 Docking Simulations of Novel FXR Ligands

We generated three focused libraries (G1, G2, and G3), each containing 1,000 unique molecules produced by our FXR fine-tuned model. These libraries were evaluated by molecular docking against the recently released X-ray structure of FXR bound to an agonist (PDB code: 3DCT65).

Molecular docking is particularly suited for validating *de novo* designs because (1) bioactivity is often not fully captured through classical molecular descriptors and QSAR models, and (2) docking relies on physicochemical complementarity to the receptor binding pocket rather than ligand similarity. As emphasized by Sattarov et al., docking provides an orthogonal validation by estimating receptor–ligand affinity, thereby offering a rigorous test for generative algorithms.^73^

As a preliminary validation of our docking protocol, we used three benchmark databases (BD1, BD2, and BD3), each containing 50 known FXR actives (randomly selected from FXR-DB) and 850 decoys (molecules confirmed as inactive against FXR, retrieved from ChEMBL and the DUDE decoy set), for an actives/total ratio of 5.88%. Following an established procedure,^13^ we ranked all 900 compounds in each benchmark by computed docking score and examined enrichment in the top decile. In BD1, the percentage of actives in the first decile rose from 5.88% to 16.88% (one-sided Fisher’s exact test, *p* = 7.48 × 10^– 5^), in BD2 to 14.29% (*p* = 3.10 × 10^– 4^), and in BD3 to 17.80% (*p* = 7.49 × 10^– 5^). These enrichments confirm that the protocol reliably prioritized true FXR binders over decoys.

We next compared docking scores of FXR-DB actives against 5,000 random ChEMBL compounds. The percentage of actives in the top decile increased from 16.3% to 36.0% (*p* = 9.57 × 10^– 8 0^), confirming that the docking procedure effectively discriminated FXR binders from nonbinders. Each focused library (1,000 generated molecules +5,000 random ChEMBL molecules) was then docked, and all 6,000 compounds were ranked by score. Enrichment in the top decile increased from a baseline of 16.66% (random) to 38.87% for G1 (*p* = 1.08 × 10^– 4 9^), 46.29% for G2 (*p* = 4.99 × 10^– 6 9^), and 42.11% for G3 (*p* = 1.83 × 10^– 6 0^). These extremely low p-values (ranging from 10^– 4 9^ to 10^– 6 9^) demonstrate that the FXR fine-tuned VeGA model consistently generated molecules with predicted binding affinities superior to random ChEMBL compounds.

Figures 5 and 6 show the 2D structures and top-scoring docking poses of five representative compounds (GFX1–GFX5) from the top decile. Structurally, these GFX ligands retain key pharmacophoric features of GW4064 while incorporating diverse scaffolds and substituents that enhance novelty and chemical space exploration. All GFX compounds present a carboxylate head forming a hydrogen bond with R331 (helix 5), mimicking the interaction of endogenous bile acids and GW4064.^57^

**Figure 5:**
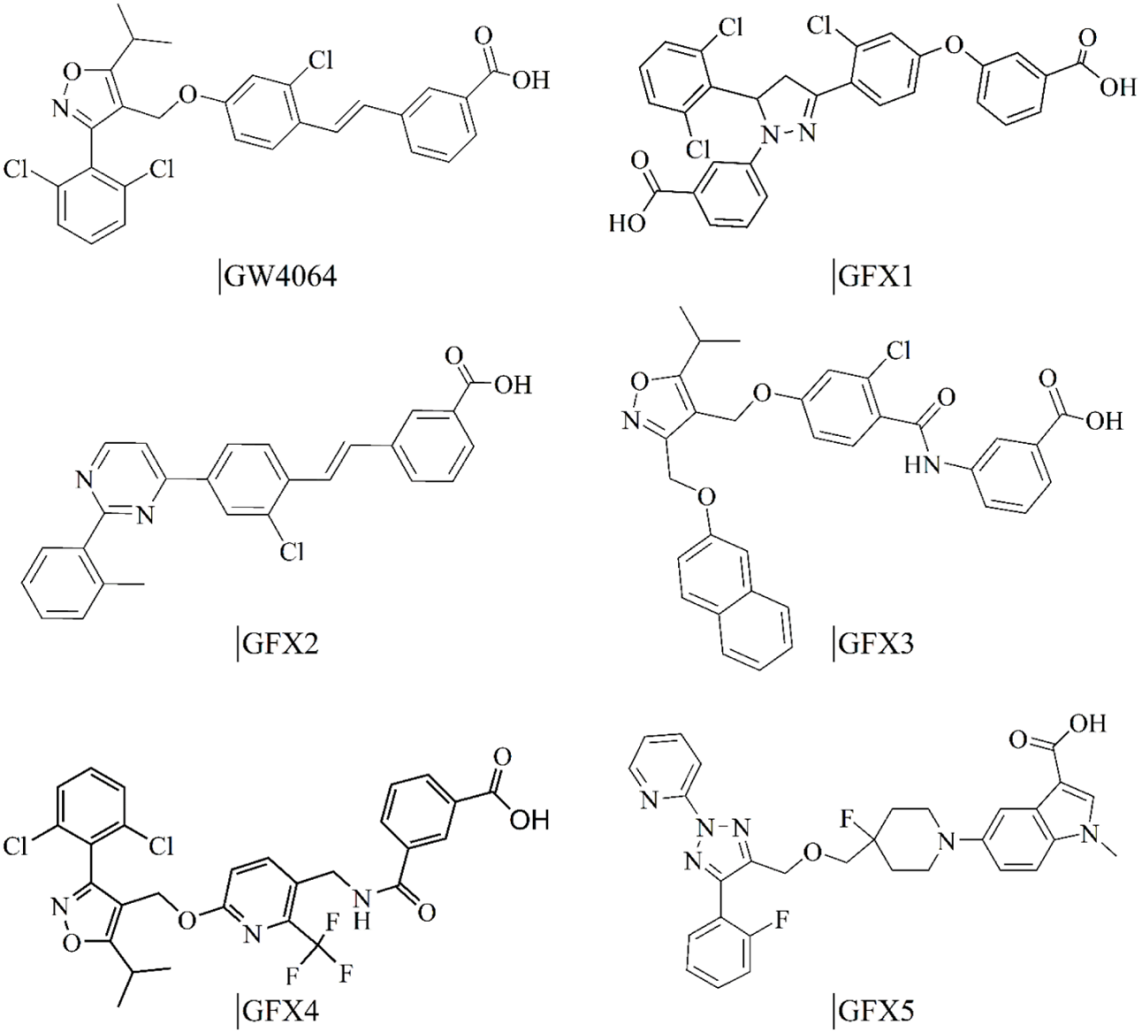
Novel chemotypes for the Farnesoid X Receptor (FXR) generated by VeGA. 2D structures of the known FXR agonist GW4064 (reference) and five representative compounds (GFX1–GFX5) generated by the FXR-tuned model. Selected from top-scoring docking results, these compounds showcase diverse and novel scaffolds while retaining essential pharmacophoric features required for binding.

**Figure 6:**
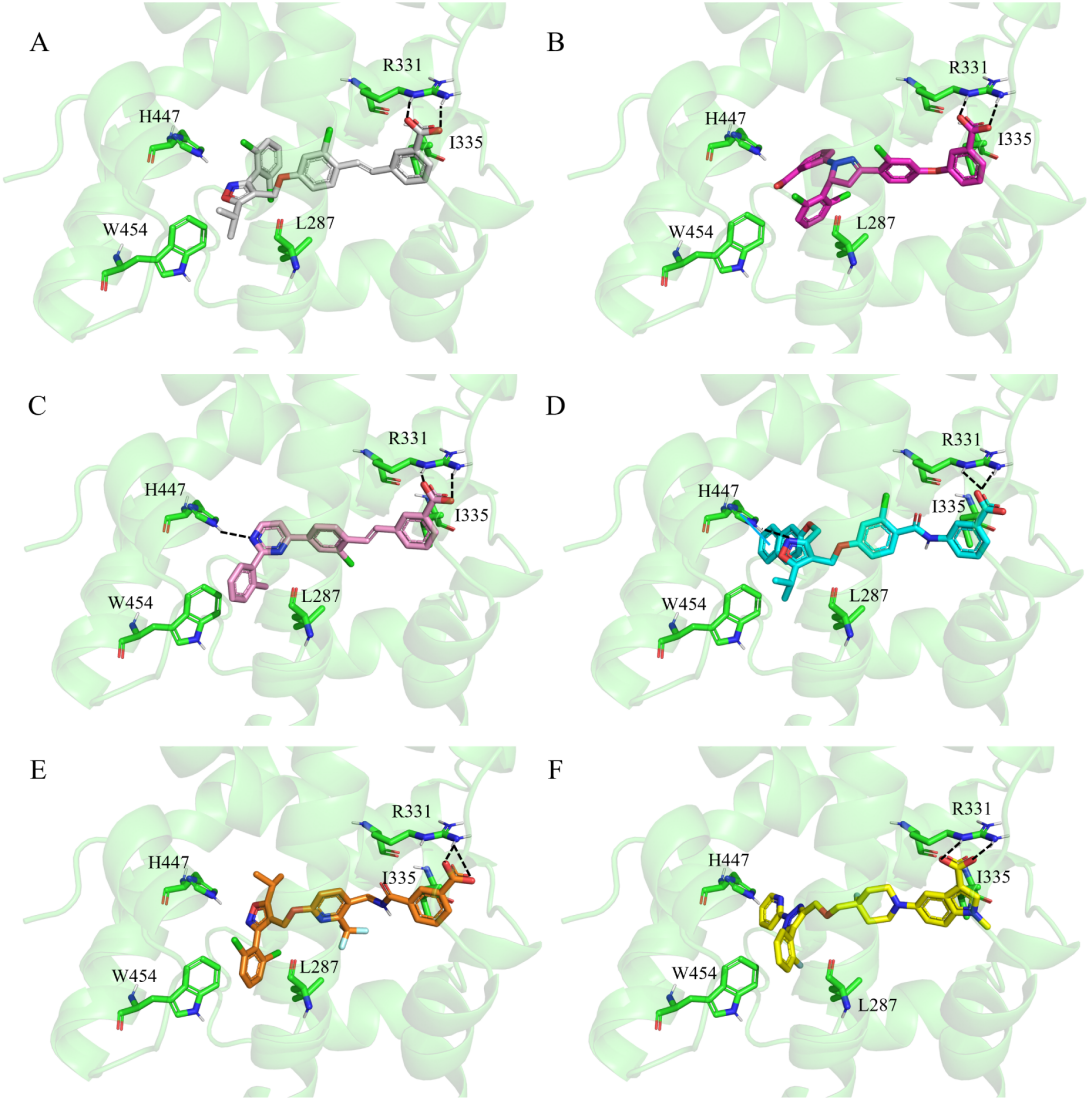
Validation of binding modes for top-scoring FXR ligands generated by VeGA. (A) Co-crystallized GW4064 in the FXR binding pocket (PDB: 3DCT). (B–F) Predicted binding poses for GFX1–GFX5. Despite their structural novelty, the ligands exhibit a conserved binding mode, replicating key interactions such as the hydrogen bond with R331 and hydrophobic contacts with L287, H447, and W454. Important residues are shown as sticks, the protein as a cartoon. Hydrogen bonds are depicted as dashed black lines, and π–π interactions as blue lines. Only polar hydrogen atoms are shown for clarity.

Importantly, the generated molecules preserved the pharmacophoric distance between the acidic head and the isoxazole moiety observed in GW4064, ensuring optimal fit in the binding cavity. In GW4064, the 3-phenyl-5-isopropyl isoxazole ring engages hydrophobic contacts with W454 and H447 (helix 10) and with L287 (helix 5). Despite diverse core structures, GFX1–GFX5 reproduced these critical hydrophobic interactions. The binding modes of all GFX compounds overlapped closely with that of GW4064 and achieved excellent docking scores (−12.45 to – 14.30 kcal/mol).

To rigorously assess novelty, we performed a systematic similarity search to identify each compound’s nearest neighbor in the FXR training database. This approach is more informative than simple identity checks for demonstrating true novelty. Results are summarized in Figure S5.

The analysis shows that top compounds displayed varying similarities to their nearest neighbors. Compounds like GFX-3 (Tanimoto = 0.34) and GFX-1 (Tanimoto = 0.44) demonstrate VeGA’s ability to generate truly novel chemotypes, whereas compounds such as GFX-5 (Tanimoto = 0.75) illustrate effective interpolation, generating close analogues that may be useful for lead optimization. Together, these findings offer strong quantitative evidence that VeGA can perform both meaningful exploration and targeted exploitation. While docking provides initial insights into binding affinity, future work will include in vitro assays to validate biological activity.

## 4 Conclusion

We have introduced VeGA, a lightweight and efficient decoder-only Transformer model for *de novo* drug-like molecule generation. Our approach balances architectural simplicity with robust performance, showing competitive results against state-of-the-art models in benchmarks such as MOSES and in large-scale unconditional generation. The true strength of VeGA emerges in challenging, target-specific applications. Our rigorous holdout-based evaluation across five pharmacological targets depicts VeGA as a powerful explorer. While other models may excel in interpolation, VeGA consistently generates the most novel molecules, highlighting its ability to balance discovery with exploration, particularly in data-scarce settings. The FXR case study further confirmed its capacity to produce novel, pharmacologically relevant compounds with validated binding potential. Despite these promising results, some limitations point to future directions. A key simplification in our current work is the exclusion of stereochemistry. We acknowledge this as significant for drug discovery, where chirality strongly influences biological activity.^74^ This choice was intentional for this foundational study, focusing on validating the core architecture’s ability to learn molecular grammar. Additionally, VeGA currently lacks explicit conditioning mechanisms. Future work will extend the architecture to enable goal-directed generation for multiobjective optimization.^33^ Beyond the specific targets examined, the potential impact of VeGA is broader. By providing a computationally accessible and highly exploratory framework, VeGA represents a valuable tool for medicinal chemists, particularly in discovery campaigns where the priority is the identification of genuinely novel scaffolds and intellectual property.

## Supplementary Material



## Data Availability

The ChEMBL database (https://www.ebi.ac.uk/chembl/) is a public domain data resource. RDKit is available at https://zenodo.org/records/15605628. Tensorflow is available at https://www.tensorflow.org/. Schrödinger Suite (https://www.schrodinger.com), a licensed software for biomolecular simulation and analysis, was used for docking studies. PyMOL (https://pymol.org/), a molecular visualization tool distributed under a license, was used for displaying and analyzing 3D structures and for figure preparation. The code, data sets and fine-tuned models are freely available under the MIT license at GitHub: https://github.com/piedelre93/VeGA-for-de-novo-design.

## Notes

Figure S1: summary of the optimized hyperparameters for VeGA. The figure shows the search ranges and steps used in the Bayesian optimization process with Optuna. The table details the parameters explored, while the graph illustrates optimization results. The red point indicates the minimum validation loss (1.08), corresponding to the final selected parameters: embedding dimension = 100, transformer layers = 4, multihead attention units = 4, dropout rate = 0.15, FFN dimension = 300, and training batch size = 64. Figure S2: overview of the developed generative model architecture. Figure S3: comparison between VeGA-generated molecules (left column) and their most similar ChEMBL counterparts (middle column). The right column shows the Tanimoto similarity coefficient for each pair, calculated using ECFP4 fingerprints. Figure S4: visual confirmation of VeGA’s explorative capability via UMAP chemical space projections. The panels display the 2D chemical space for five fine-tuned targets: (A) FXR, (B) GBA, (C) MAPK1, (D) PKM2, and (E) mTORC1. In each plot, the training set (faint gray dots) and unseen holdout actives (green stars) are shown relative to the large cloud of VeGA-generated molecules (blue circles). The consistent envelopment of holdout actives by the generated molecules provides strong visual evidence of VeGA’s ability to generalize and discover novel pharmacologically relevant regions of chemical space. Figure S5: comparison between VeGA-generated molecules (left column) and their most similar FXR ligands (middle column). The right column shows the Tanimoto similarity coefficient for each pair, calculated using ECFP4 fingerprints. Table S1: parameters used during training. Table S2: systematic analysis of the effect of SMILES augmentation probability on model performance. The table reports key metrics, including validity, Fréchet ChemNet Distance (FCD/Test), and internal diversity (IntDiv, IntDiv2), for VeGA models trained with augmentation levels from 0% to 50%. The results highlight a trade-off between chemical realism (FCD, lower is better) and structural diversity (higher IntDiv). A 10% augmentation rate was selected as the optimal compromise, providing increased diversity with only a marginal decrease in fidelity. Table S3: statistical measures (mean, median, standard deviation, minimum, and maximum values) for physicochemical properties relevant to drug development. Properties include molecular weight, lipophilicity (LogP), drug-likeness (QED), synthetic accessibility (SA), hydrogen bond donors/acceptors, rotatable bonds, and ring count. Calculations were performed on 1,092,285 ChEMBL-DB molecules and 100,000 molecules generated by VeGA, R4, and S4 using RDKit.Table S4. Statistical comparison between ChEMBL-DB and VeGA-generated molecules. The upper section reports similarity between property distributions using the Kolmogorov–Smirnov (KS) statistic and Kullback–Leibler (KL) divergence. The lower section reports compliance percentages with drug-likeness filters: Lipinski’s Rule of 5 (MW ≤ 500, logP ≤ 5, HBD ≤ 5, HBA ≤ 10) and PAINS substructure absence. Analysis was performed with RDKit on 100,000 molecules generated by VeGA, R4, and S4, compared with 1,092,285 ChEMBL-DB compounds.Table S5: comparative analysis of VeGA, S4, R4, TRACER, LLaMol, and MolGPT models, detailing their architectural, computational, and design characteristics. The table provides a side-by-side comparison of each model’s core architecture, number of trainable parameters, pretraining data set (including version and size), and hardware requirements. It specifically highlights the key differences in computational cost, contrasting the hyperparameter search duration (e.g., ∼36 h on a single GPU for VeGA vs ∼10 days on multiple GPUs for S4), pretraining time, and inference speed. All information for the S4 and R4 baseline models was compiled from their respective original publications where available; fields marked “Not Reported” indicate data not provided in the source literature. Table S6: comparison of Token Vocabularies from ChEMBL-DB and COCONUT Data sets. Table S7: architectural hyperparameters for the original VeGA model and a deeper variant developed for NP generation. To test whether increased representational power improves performance on the structurally complex NP data set, the deeper model doubled several capacity-related parameters, including embedding dimension, number of Transformer layers and attention heads, and FFN dimension. Table S8: statistical measures (mean, median, standard deviation, minimum, and maximum) for physicochemical properties relevant to NPs, including fraction of sp^3^ carbons, aliphatic rings, spiro atoms, heavy atoms, molecular weight, and largest fused ring system size. Calculations were performed on 32,360 ChEMBL-DB molecules and 100,000 VeGA-generated molecules using RDKit.Table S9. Statistical comparison between COCONUT and VeGA-generated molecules using the KS statistic, ranging from 0 (identical distributions) to 1 (completely different) (PDF)
