# Supplementary material for: VeGA: A Versatile Generative Architecture for Bioactive Molecules across Multiple Therapeutic Targets
Source: J Chem Inf Model. 2025 Oct 2;65(20):10918–31. doi: 10.1021/acs.jcim.5c01606 (PMC12570142; doi:10.1021/acs.jcim.5c01606)
Supplement: Supplementary file 1 [file ci5c01606_si_001.pdf]

Supporting Information for:

VeGA: A Versatile Generative Architecture for Bioactive Molecules Across Multiple Therapeutic Targets

Pietro Delre[a], Antonio Lavecchia[a]*

[a]*Department of Pharmacy, "Drug Discovery Laboratory", University of Naples Federico II, via Domenico Montesano 49, I-80131 Naples, Italy*;

*Email: antonio.lavecchia@unina.it

Supplementary material - Table of content

Figure S1. Summary of the optimized hyperparameters for VeGA. The figure shows the search ranges and steps used in the Bayesian optimization process with Optuna. The table details the parameters explored, while the graph illustrates optimization results. The red point indicates the minimum validation loss (1.08), corresponding to the final selected parameters: embedding dimension = 100, Transformer layers = 4, multi-head attention units = 4, dropout rate = 0.15, FFN dimension = 300, and training batch size = 64.

Figure S2. Overview of the developed generative model architecture.

Figure S3. Comparison between VeGA-generated molecules (left column) and their most similar ChEMBL counterparts (middle column). The right column shows the Tanimoto similarity coefficient for each pair, calculated using ECFP4 fingerprints.

Figure S4. Visual confirmation of VeGA's explorative capability via UMAP chemical space projections. The panels display the 2D chemical space for five fine-tuned targets: (A) FXR, (B) GBA, (C) MAPK1, (D) PKM2, and (E) mTORC1. In each plot, the training set (faint grey dots) and unseen holdout actives (green stars) are shown relative to the large cloud of VeGA-generated molecules (blue circles). The consistent envelopment of holdout actives by the generated molecules provides strong visual evidence of VeGA's ability to generalize and discover novel pharmacologically relevant regions of chemical space.

Figure S5. Comparison between VeGA-generated molecules (left column) and their most similar FXR ligands (middle column). The right column shows the Tanimoto similarity coefficient for each pair, calculated using ECFP4 fingerprints.

Table S1. Parameters used during training.

Table S2. Systematic analysis of the effect of SMILES augmentation probability on model performance. The table reports key metrics, including validity, Fréchet ChemNet Distance

(FCD/Test), and internal diversity (IntDiv, IntDiv2), for VeGA models trained with augmentation levels from 0% to 50%. The results highlight a trade-off between chemical realism (FCD, lower is better) and structural diversity (higher IntDiv). A 10% augmentation rate was selected as the optimal compromise, providing increased diversity with only a marginal decrease in fidelity.

Table S3. Statistical measures (mean, median, standard deviation, minimum, and maximum values) for physicochemical properties relevant to drug development. Properties include molecular weight, lipophilicity (LogP), drug-likeness (QED), synthetic accessibility (SA), hydrogen bond donors/acceptors, rotatable bonds, and ring count. Calculations were performed on 1,092,285 ChEMBL-DB molecules and 100,000 molecules generated by VeGA, R4, and S4 using RDKit.

Table S4. Statistical comparison between ChEMBL-DB and VeGA-generated molecules. The upper section reports similarity between property distributions using the Kolmogorov–Smirnov (KS) statistic and Kullback–Leibler (KL) divergence. The lower section reports compliance percentages with drug-likeness filters: Lipinski's Rule of 5 (MW ≤ 500, logP ≤ 5, HBD ≤ 5, HBA ≤ 10) and PAINS substructure absence. Analysis was performed with RDKit on 100,000 molecules generated by VeGA, R4, and S4, compared with 1,092,285 ChEMBL-DB compounds.

Table S5. Comparative analysis of VeGA, S4, R4, TRACER, LLaMol, and MolGPT models, including architecture, number of trainable parameters, pre-training dataset (version and size), and hardware requirements. The table highlights differences in computational cost, such as hyperparameter search time (~36 h on a single GPU for VeGA vs. ~10 days on multiple GPUs for S4), pre-training duration, and inference speed. Information for models was obtained from

their original publications; fields marked "Not Reported in Publication" indicate unavailable data.

Table S6. Comparison of Token Vocabularies from ChEMBL-DB and COCONUT Datasets.

Table S7. Architectural hyperparameters for the original VeGA model and a deeper variant developed for natural product (NP) generation. To test whether increased representational power improves performance on the structurally complex NP dataset, the deeper model doubled several capacity-related parameters, including embedding dimension, number of Transformer layers and attention heads, and FFN dimension.

Table S8. Statistical measures (mean, median, standard deviation, minimum, and maximum) for physicochemical properties relevant to natural products, including fraction of sp^3 carbons, aliphatic rings, spiro atoms, heavy atoms, molecular weight, and largest fused ring system size. Calculations were performed on 32,360 ChEMBL-DB molecules and 100,000 VeGA-generated molecules using RDKit.

Table S9. Statistical comparison between COCONUT and VeGA-generated molecules using the KS statistic, ranging from 0 (identical distributions) to 1 (completely different).

Parameter	Search space	Step
Embedding dimensionality	[80–100]	20
Number of Transformer layers	[1–4]	1
Number of multi-head attention units	[1–4]	1
Dropout rate	[0.1–0.3]	0.05
FFN dimension	[100–500]	100
Training batch size	[32, 64, 128]	

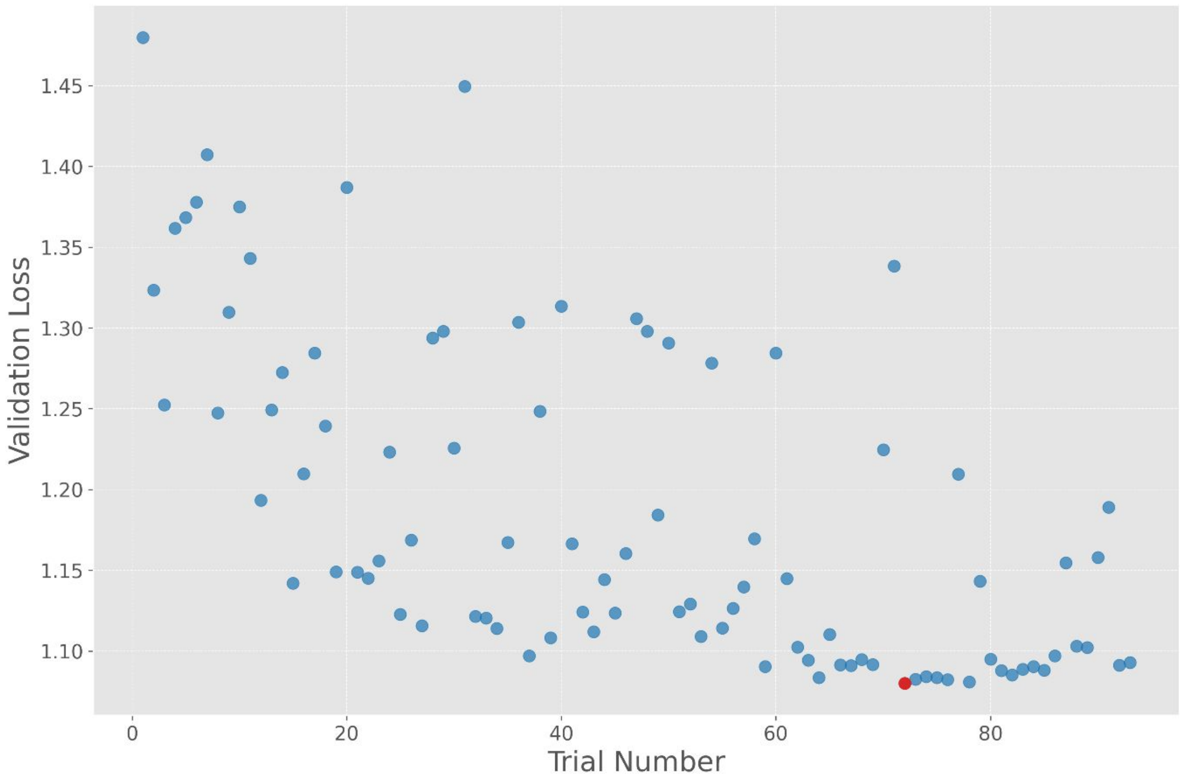

Figure S1. Summary of the optimized hyperparameters for VeGA, including their search ranges and steps utilized in the Bayesian optimization process with Optuna. The table details the parameters explored, while the graph illustrates the optimization trial results, with the red point indicating the minimum validation loss of 1.08, corresponding to the parameters: embedding dimensionality = 100, Number of Transformer layers = 4, Number of multi-head attention units: 4, Dropout rate = 0.15, FFN dimension = 300 and training batch size = 64

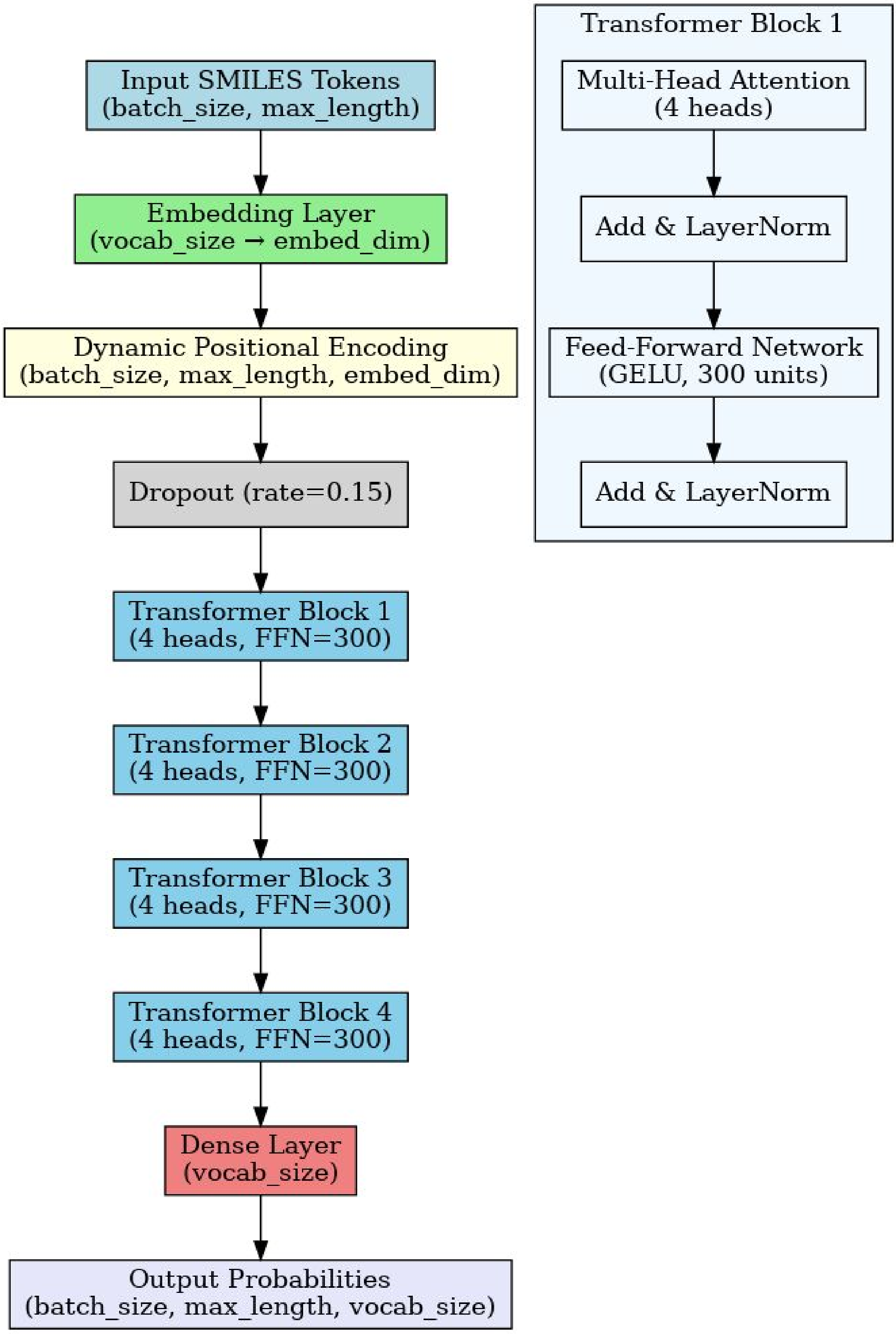

Figure S2. Detailed overview of the developed generative model architecture.

VeGA-generated molecules	ChEMBL similar molecules	Similarity
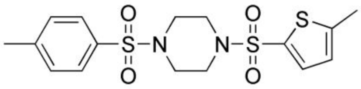<br>26544380	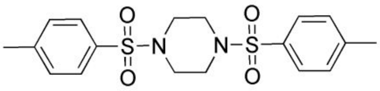<br>CHEMBL596080	68.75%
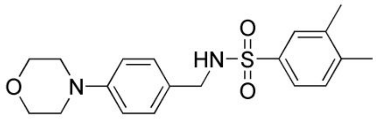<br>52327650	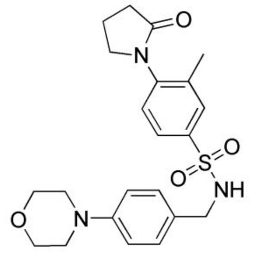<br>CHEMBL4922096	69.09%
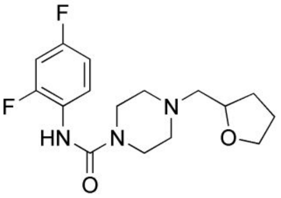<br>51168649	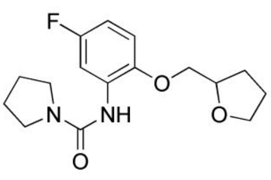<br>CHEMBL3494097	60.34%
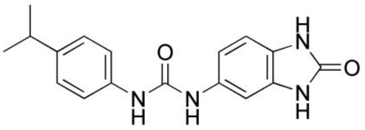<br>48741373	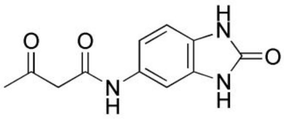<br>CHEMBL3187749	55.81%
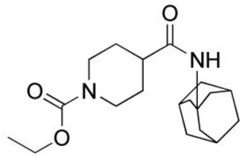<br>26910669	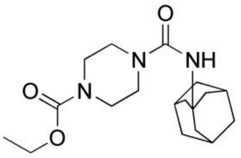<br>CHEMBL1378603	69.05%
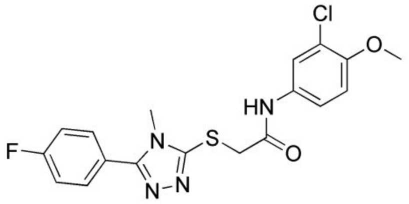<br>8112638	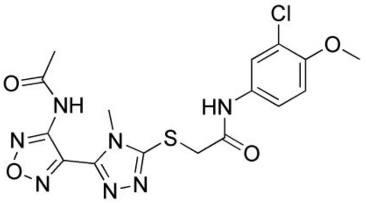<br>CHEMBL1434965	65.22%
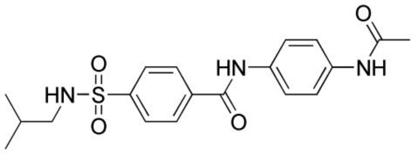<br>109058979	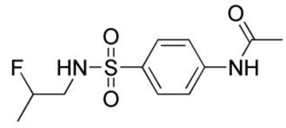<br>CHEMBL2333971	67.44%
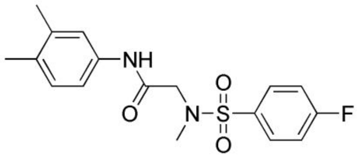<br>4798013	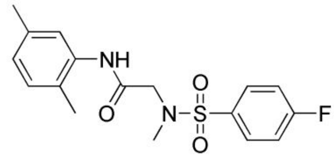<br>CHEMBL1404449	69.39%

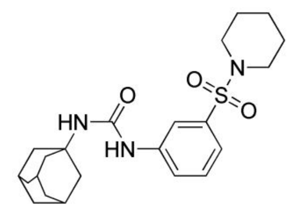 4803265	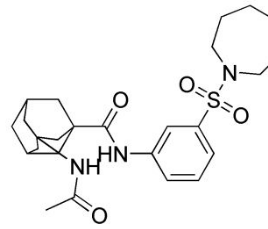 CHEMBL1875197	65.57%
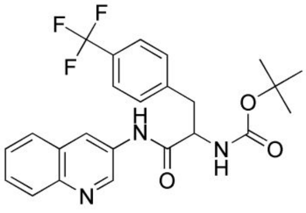 77391424	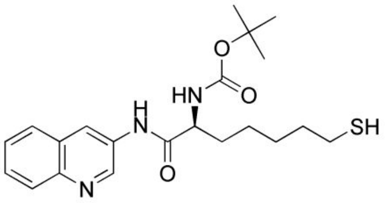 CHEMBL235263	64.18%

Figure S3. Comparison between VeGA-generated molecules (left column) and their most similar ChEMBL counterparts (middle column). The rightmost column shows the Tanimoto similarity coefficient between each pair, calculated using ECFP4 fingerprints.

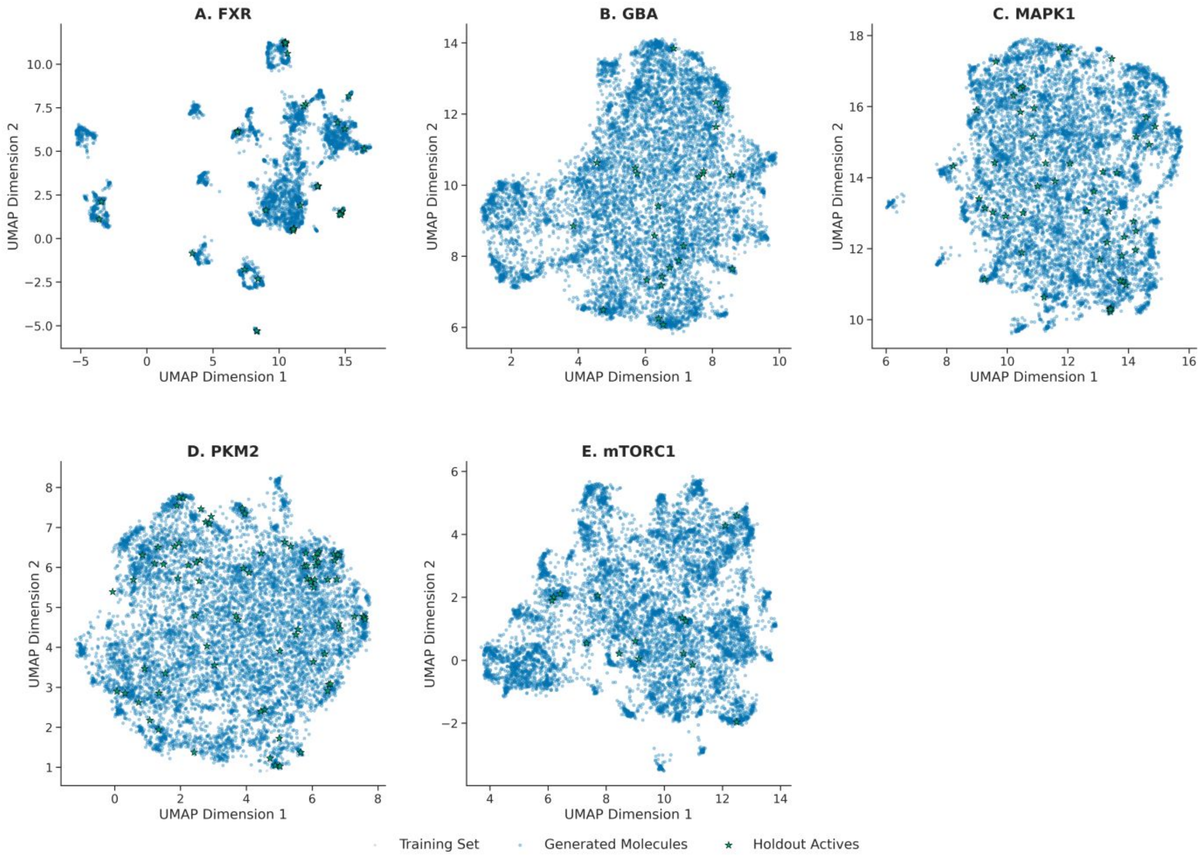

Figure S4. Visual confirmation of VeGA's explorative capability via UMAP chemical space projections. The panels display the 2D chemical space for all five fine-tuned targets: (A) FXR, (B) GBA, (C) MAPK1, (D) PKM2, and (E) mTORC1. In each plot, the training set (faint grey dots) and the unseen holdout actives (green stars) are shown in relation to the large cloud of molecules generated by VeGA (blue circles). The consistent envelopment of the holdout actives by the generated cloud provides strong visual evidence of the model's ability to learn from the training data and generalize to discover novel, pharmacologically relevant regions of chemical space.

VeGA-generated molecules	ChEMBL similar molecules	TS to Nearest Neighbor
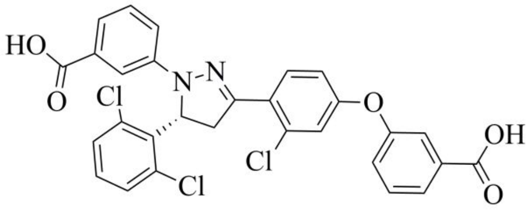 GFX1	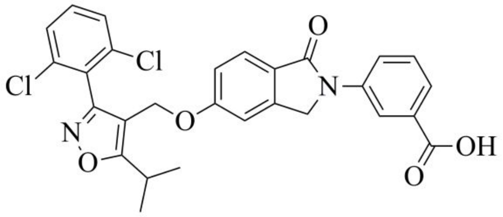 CHEMBL564917	0.34
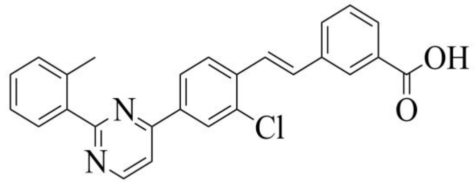 GFX2	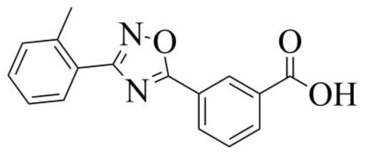 CHEMBL3589975	0.44
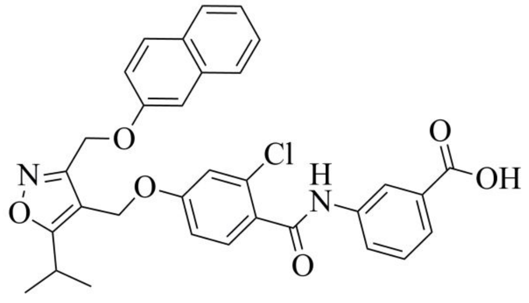 GFX3	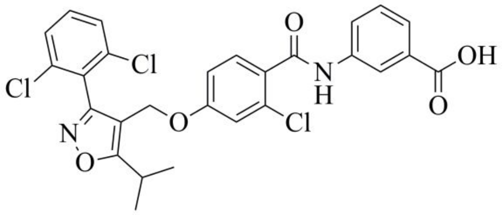 CHEMBL389998	0.75
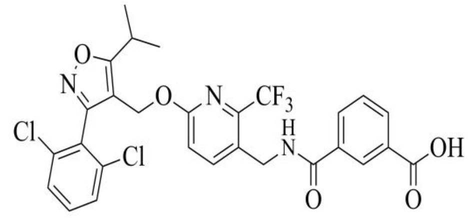 GFX4	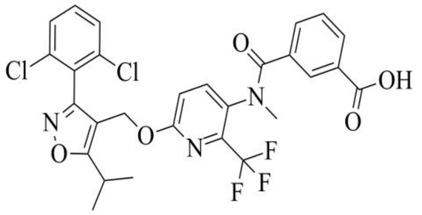 CHEMBL1209058	0.71
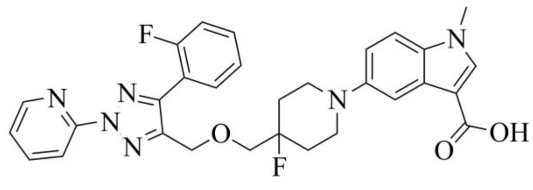 GFX5	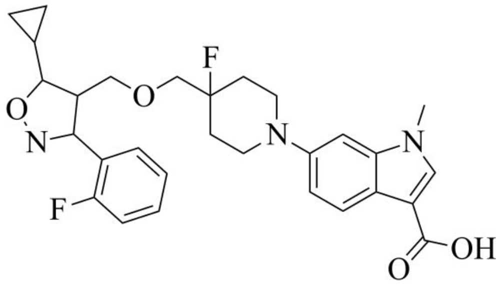 CHEMBL4800066	0.59

Figure S5. Comparison between VeGA-generated molecules (left column) and their most similar FXR ligand counterparts (middle column). The rightmost column shows the Tanimoto similarity coefficient between each pair, calculated using ECFP4 fingerprints.

Table S1. List of parameters employed during training phase.

**Parameter**	**Value**	**Description**
Batch_size	64	Number of samples processed in one forward/backward pass during training.
Max_lenght	(inferred from SMILES)	Maximum allowed length for SMILES sequences in the dataset.
Vocab_size	(inferred from SMILES)	Number of unique tokens in the generated vocabulary.
Embed_dim	100	Dimension of the token embeddings in the model.
Dropout_rate	0.15	Probability of randomly dropping neurons during training to prevent overfitting.
Epochs	300	Number of complete passes through the training dataset.
Transformer_Heads	4	Number of attention heads in the multi-head self-attention mechanism.
Transformer_Layers	4	Number of stacked Transformer encoder/decoder layers.
FF_Dim	300	Dimension of the feed-forward network (FFN) inside the Transformer layers.
Valid_Ratio	0.2	Fraction of the dataset reserved for validation (10%).
Temperature	1	Initial temperature parameter for sampling during generation (controls randomness).
Temperature_Decay	0.97	Factor by which temperature is reduced over time to make sampling more deterministic.
Warmup_Epochs	50	Number of epochs during which curriculum learning gradually increases complexity.

L2_REG	1e-5	L2 regularization coefficient to penalize large weights and prevent overfitting.
Augment_Prob	0.1	Increment step for increasing complexity in curriculum learning.

Table S2. Systematic analysis of the impact of SMILES augmentation probability on generative model performance. The table shows key metrics, including Validity, Fréchet ChemNet Distance (FCD/Test), and Internal Diversity (IntDiv, IntDiv2), for VeGA models trained with varying levels of randomized SMILES augmentation (from 0% to 50%). The results highlight a clear trade-off between chemical realism (FCD, lower is better) and structural diversity (Internal Diversity, higher is better). A 10% augmentation rate was selected as the optimal compromise, as it provides a beneficial increase in diversity for only a marginal decrease in distributional fidelity.

**Model (Augmentation %)**	**Valid**	**Unique@1k**	**Unique@10k**	**FCD/Test**	**FCD/TestSF**	**IntDiv**	**IntDiv2**
**AUG-0 (0%)**	0.97	100.00	100.00	**0.18**	0.55	**0.85**	0.85
**AUG-01 (10%)**	0.97	100.00	100.00	**0.20**	0.56	**0.86**	0.85
**AUG-02 (20%)**	0.96	100.00	100.00	**0.30**	0.87	**0.86**	0.85
**AUG-03 (30%)**	0.97	100.00	100.00	**0.67**	1.29	**0.86**	0.86
**AUG-04 (40%)**	0.97	100.00	100.00	**1.03**	1.65	**0.86**	0.86
**AUG-05 (50%)**	0.95	100.00	100.00	**1.56**	2.31	**0.87**	0.86

Table S3. This table presents detailed statistical measures (mean, median, standard deviation, minimum, and maximum values) for physicochemical properties relevant to drug development. Properties include molecular weight, lipophilicity (LogP), drug-likeness (QED), synthetic accessibility (SA), hydrogen bond donors and acceptors, rotatable bonds, and ring count. Statistics were calculated from 1,092,285 ChEMBL-DB molecules and 100,000 VeGA, R4 and S4 generated molecules using RDKit.

Property	Statistical Measure	ChEMBL-DB	VeGA	R4	S4
**Molecular Weight (Da)**	Mean	405.41	403.95	383.84	397.30
	Median	392.50	395.47	375.43	387.39
	Standard Deviation	107.24	103.60	102.47	106.22
	Minimum	200.08	62.00	73.10	31.99
	Maximum	804.08	949.38	1141.09	1360.89
**LogP**	Mean	3.59	3.65	3.40	3.36
	Median	3.57	3.63	3.37	3.35
	Standard Deviation	1.82	1.86	1.75	1.83
	Minimum	-11.07	-6.66	-11.32	-11.54
	Maximum	18.00	16.05	17.51	19.12
**QED Score**	Mean	0.55	0.54	0.58	0.57
	Median	0.56	0.55	0.60	0.59
	Standard Deviation	0.22	0.21	0.21	0.21
	Minimum	0.01	0.01	0.01	0.01
	Maximum	0.95	0.95	0.95	0.95
**SA Score**	Mean	2.93	2.77	2.91	2.84
	Median	2.74	2.72	2.76	2.66
	Standard Deviation	0.82	0.76	0.79	0.76
	Minimum	1.06	1.14	1.00	1.05
	Maximum	8.29	7.96	7.97	7.99

**H-Bond Donors**	Mean	1.56	1.49	1.47	1.45
	Median	1.00	1.00	1.00	1.00
	Standard Deviation	1.41	1.30	1.31	1.41
	Minimum	0.00	0.00	0.00	0.00
	Maximum	21.00	14.00	17.00	17.00
**H-Bond Acceptors**	Mean	5.25	5.23	4.80	5.36
	Median	5.00	5.00	5.00	5.00
	Standard Deviation	2.21	2.15	1.98	2.21
	Minimum	0.00	0.00	0.00	0.00
	Maximum	24.00	22.00	26.00	26.00
**Rotatable Bonds**	Mean	5.74	5.72	5.26	5.46
	Median	5.00	5.00	5.00	5.00
	Standard Deviation	3.44	3.48	3.24	3.43
	Minimum	0.00	0.00	0.00	0.00
	Maximum	51.00	50.00	52.00	86.00
**Ring Count**	Mean	3.56	3.52	3.37	3.58
	Median	3.00	3.00	3.00	4.00
	Standard Deviation	1.33	1.25	1.26	1.28
	Minimum	0.00	0.00	0.00	0.00
	Maximum	23.00	10.00	14.00	15.00

Table S4. Comprehensive statistical comparison between ChEMBL-DB and VeGA-generated molecules. The upper section shows the similarity between property distributions using the Kolmogorov-Smirnov (KS) statistic and the Kullback-Leibler (KL) Divergence. The lower section displays compliance percentages with drug-likeness filters: Lipinski's Rule of 5 (MW ≤ 500, logP ≤ 5, HBD ≤ 5, HBA ≤ 10) and PAINS (Pan Assay Interference Compounds) substructure absence. Analysis performed using RDKit on 100,000 VeGA, R4 and S4 generated molecules compared against 1,092,285 ChEMBL-DB compounds.

**Property**	*KS test*			*KL test*		
	VeGA	***R4***	***S4***	***VEGA***	***R4***	***S4***
**Molecular Weight**	0.0148	0.0734	0.0252	0.0906	0.1916	0.1711
**LogP**	0.0226	0.0512	0.0561	0.0031	0.0085	0.0102
**QED Score**	0.0114	0.0640	0.0456	0.0022	0.0114	0.0069
**SA Score**	0.0210	0.0201	0.0515	0.0078	0.0088	0.0078
**H-Bond Donors**	0.0122	0.0198	0.0480	0.0024	0.0023	0.0063
**H-Bond Acceptors**	0.0097	0.0822	0.0248	0.0024	0.0240	0.0020
**Rotatable Bonds**	0.0058	0.0657	0.0475	0.0012	0.0129	0.0069
**Ring Count**	0.0117	0.0584	0.0153	0.0042	0.0107	0.0023
	**Medicinal Chemistry Filter Compliance (%)**					
	**CHEMBL-DB**		***VeGA***		***R4***	***S4***
**Lipinski's Rule of 5**	70.37		68.24		72.24	70.41
**PAINS-free**	93.60		94.85		93.85	94.45

Table S5. Comparative analysis of VeGA, S4, R4, TRACER, LLaMol, and MolGPT models, detailing their architectural, computational, and design characteristics. The table provides a side-by-side comparison of each model's core architecture, number of trainable parameters, pre-training dataset (including version and size), and hardware requirements. It specifically highlights the key differences in computational cost, contrasting the hyperparameter search duration (e.g., ~36 hours on a single GPU for VeGA vs. ~10 days on multiple GPUs for S4), pre-training time, and inference speed. All information for the S4 and R4 baseline models was compiled from their respective original publications where available; fields marked "Not Reported" indicate data not provided in the source literature.

Metric	VeGA	S4	R4	TRACER	LLaMol	MolGPT
**Core Architecture**	Decoder-only Transformer	Structured State Space Sequence (S4)	RNN (LSTM) / Transformer Hybrid	Conditional Transformer + GCN + Monte Carlo Tree Search (MCTS)	Transformer inspired by LLaMA-2	Decoder-only Transformer
**Trainable Parameters**	~0.8 M (806,443)	Not Reported	Not Reported	~45 M *(estimated from architecture: 6 encoder + 6 decoder layers, d_model=512, 8 heads, FFN=2048)*	~15 M	~8.6 M
**Pre-training Dataset**	ChEMBL v28 (~1.1 M compounds)	ChEMBL v31 (~1.9 M compounds)	ChEMBL (v25–28) (~1.7 M compounds)	USPTO-1k TPL (~445 K reactions)	OrganiX13 (ZINC15, ChEMBL, PubChemQC; ~12.5 M compounds)	GuacaMol (~1.9 M compounds)
**Hyperparameter Search**	~36 h (Bayesian search, 100 trials, 20 K samples)	~10 days on multiple GPUs	Not Reported	Optuna for GCN (details not reported)	Not Reported	Not Reported

**Pre-training Time**	~7 h for 50 epochs	>1.3× faster than LSTM (exact time not reported)	Not Reported	Not Reported	~48 h	Not Reported *(10 epochs)*
**Fine-tuning Protocol**	Max 100 epochs with early stopping	Not Reported	Not Reported	MCTS with QSAR models as reward (200 steps, beam width 10–50)	Not Reported	Conditional generation (molecular properties and/or scaffolds)
**Hardware (Primary)**	Single NVIDIA RTX A2000 (12 GB VRAM)	Multiple NVIDIA A100 (40 GB VRAM)	Not Reported	Single NVIDIA RTX 4090 (24 GB VRAM)	NVIDIA A100 (40 GB VRAM)	NVIDIA RTX 2080 Ti (11 GB VRAM)
**Inference Time (per molecule, batch of 1000)**	~20 ms	Not Reported	Not Reported	Not Reported	Not Reported	Not Reported
**Design Philosophy**	Lightweight, computationally efficient, “explorer”	High-performance, long-range dependency modeling	Modular, open-source, versatile	Reaction-aware generative design	Flexible, multi-conditional generation (4+ conditions)	Focus on multi-property and scaffold-based conditional generation

Table S6. Comparison of Token Vocabularies from ChEMBL-DB and COCONUT Datasets.

Token Category	COCONUT Vocabulary	ChEMBL Vocabulary	Description
**Special Tokens**	<PAD>, <START>, <END>	<PAD>, <START>, <END>	Padding, sequence start/end markers
**Ring Closures**	1, 2, 3, 4, 5, 6, 7, 8, 9, %10, %11, %12, %13, %14, %15	0, 1, 2, 3, 4, 5, 6, 7, 8, 9	Ring closure digits
**Branches**	(,)	(,)	Parentheses for branching
**Bonds**	#, =	#, =	Bond types (single, double, triple)
**Atoms**	B, Br, C, Cl, F, I, N, O, P, S	Br, C, Cl, F, I, N, O, P, S	Atomic symbols
**Charged Atoms**	[B-], [C+], [C-], [CH-], [CH2-], [CH], [C], [N+], [N-], [NH+], [NH-], [NH2+2], [NH2+], [NH3+], [N], [O-], [OH+], [O], [P+], [PH], [S+], [SH]	[C+], [C-], [CH-], [I+], [N+], [N-], [O+], [O-], [P+], [PH], [S+], [S]	Atoms with formal charges

Table S7. Architectural hyperparameter configurations for the original VeGA model and the deeper variant developed for the natural product (NP) generation experiment. To test the hypothesis that increased representational power would improve performance on the structurally complex NP dataset, a deeper model was implemented with a doubling of key capacity-related parameters, including the embedding dimension, the number of Transformer layers and attention heads, and the dimensionality of the feed-forward network.

Hyperparameter	Original VeGA	Deeper VeGA (for NP)
Embedding Dimension	100	200
Transformer Layers	4	8
Attention Heads	4	8
Feed-Forward Dimension	300	600

Table S8. This table presents detailed statistical measures (mean, median, standard deviation, minimum, and maximum values) for physicochemical properties relevant to natural products. Properties include the fraction of sp3-hybridized carbon atoms, aliphatic rings, spiro atoms, and heavy atoms, as well as the molecular weight and the size of the largest fused ring system. Statistics were calculated from 32,360 ChEMBL-DB molecules and 100,000 VeGA-generated molecules using RDKit.

Property	Statistical Measure	COCONUT	VeGA Generated
**Molecular Weight (Da)**	Mean	1061.42	1080.49
	Median	990.32	1056.44
	Standard Deviation	295.45	230.78
	Minimum	343.36	125.15
	Maximum	3346.75	2695.20
**Heavy Atoms**	Mean	75.19	75.76
	Median	70.00	74.00
	Standard Deviation	20.34	15.65
	Minimum	26.00	9.00
	Maximum	209.00	192.00
**NP-likeness Score**	Mean	1.59	1.58
	Median	1.70	1.64
	Standard Deviation	0.71	0.70
	Minimum	-0.86	-0.48
	Maximum	3.51	3.64
**Fraction sp^3 (Fsp^3)**	Mean	0.67	0.78
	Median	0.68	0.91
	Standard Deviation	0.23	0.22
	Minimum	0.00	0.00
	Maximum	1.00	1.00

**Aliphatic Rings**	Mean	6.48	5.94
	Median	6.00	6.00
	Standard Deviation	3.79	3.25
	Minimum	0.00	0.00
	Maximum	40.00	22.00
**Maximum Fused Ring Size**	Mean	10.69	8.81
	Median	6.00	6.00
	Standard Deviation	7.24	6.24
	Minimum	0.00	0.00
	Maximum	78.00	68.00
**Spiro Atoms**	Mean	0.60	0.40
	Median	0.30	0.30
	Standard Deviation	1.24	0.55
	Minimum	0.00	0.00
	Maximum	11.00	7.00

Table S9. Comprehensive statistical comparison between COCONUT and VeGA-generated molecules. The table shows the similarity between property distributions using the Kolmogorov-Smirnov (KS) statistic, which ranges from 0 (identical distributions) to 1 (completely different distributions).

Property	Distribution Similarity (KS statistic)
NP_likeness	0.073
fsp3	0.3052
Aliphatic_Rings	0.0710
Spiro_Atoms	0.1737
MW	0.1053
Max_Fused_Ring_Size	0.1863
Heavy_Atoms	0.0942
